# Supplementary material for: A theoretical framework for controlling complex microbial communities
Source: Nat Commun. 2019 Mar 5;10:1045. doi: 10.1038/s41467-019-08890-y (PMC6401173; doi:10.1038/s41467-019-08890-y)
Supplement: Supplementary file 1 — Supplementary Information [file 41467_2019_8890_MOESM1_ESM.pdf]

SUPPLEMENTARY NOTES

A THEORETICAL FRAMEWORK FOR CONTROLLING COMPLEX MICROBIAL COMMUNITIES

MARCO TULIO ANGULO[a], CLAUDE H. MOOG[b] and YANG-YU LIU[c,d]

CONTENTS

1 Preliminaries **2**
1.1 Mathematical models of microbial communities. 2
1.2 Modeling control actions in microbial communities 4
1.3 Controlled population dynamics with meromorphic functions. 4
2 Mathematical formalism to detect autonomous elements **6**
2.1 Notation 6
2.2 Characterizing autonomous elements in continuous control systems 6
2.3 Characterizing autonomous elements in impulsive control systems 6
3 Detecting autonomous elements without knowledge of the population dynamics . . **9**
3.1 Characterizing the generic absence of autonomous elements 9
3.2 Generic absence of autonomous elements in cGLV models 10
4 Identifying a minimal set of driver species **12**
5 Sufficient number of impulses to drive a microbial community **14**
6 Calculating control inputs for microbial communities with uncertain population dynamics or large number of species **18**
7 Simulation results using real microbial networks **20**
7.1 Simulation results for the core microbiota of Ircinia oros 20
7.2 Simulation results for the mice gut microbiota 22
8 Numerical validation in large microbial communities **25**
9 Controlling the repressilator **28**
Supplementary References **29**

Date: February 5, 2019
[a]CONACyT - Institute of Mathematics, Universidad Nacional Autónoma de México, Juriquilla 76230, México.
[b]Laboratoire des Sciences du Numérique de Nantes, UMR CNRS 6004, Nantes 44321, France.
[c]Channing Division of Network Medicine, Brigham and Women's Hospital and Harvard Medical School, Boston, Massachusetts 02115, USA.
[d]Center for Cancer Systems Biology, Dana-Farber Cancer Institute, Boston MA 02115, USA.
.

1. Preliminaries

1.1. **Mathematical models of microbial communities.** Some defining characteristics of microbial communities are:

1. *Dynamic abundance profiles of their species.* Microbial communities are dynamic ecosystems. The abundance of their species can exhibit rich temporal behavior including equilibria, periodic oscillations, and chaos [1, 2].
2. *Complex ecological networks.* Within a microbial community, the species form a complex network of ecological interactions through metabolic, physical, regulatory, and signaling exchanges [3]. This ecological network is a key attribute of microbial communities, in the sense that some species can survive only due their interactions with other species [4]. The ecological network of a microbial community can be directly computed from mono-culture and co-culture experiments [5], inferred from time-resolved abundance data using system identification techniques [6, 7], or inferred from steady-state samples that have distinct species collections [8].
3. *Spatial organization.* Microbial communities can exhibit complex spatial structures such as dense free-floating aggregates or surface-attached biofilms [3]. This happens, among other factors, due to motility and gradients of metabolites, light, and temperature.
4. *Host-microbe interactions*. Examples include immunologic processes in the gut microbiota of animals, and nutrient limitation in the rhizosphere microbiota of crops.
5. *Environmental factors*. Examples include salinity, temperature, pH, conductivity, etc.

To build a mathematical model of a microbial community, the first step is to decide which of its defining characteristics should be considered in the model. Such decision is summarized by defining the *state* of the community. The second step is then to select which level of details should be considered in the model. Different combinations of the above two steps produce different modeling frameworks (see e.g. [3, 9] for recent reviews), including:

1. *Population dynamics models.* These models focus on modeling changes in size and composition of the abundance profile of a microbial community. Examples of this framework are the canonical Generalized Lotka-Volterra (GLV) equations and other models based on Ordinary Differential Equations (ODEs), see below.
2. *Individual-based models.* These models focus on individual microbes as "agents" that have a set of predefined rules determining their behavior.
3. *Stoichiometric models.* These models focus on modeling metabolites inside microbial cells. Usually, the transformation of metabolites is captured by chemical reaction networks. Examples of this framework are the static and dynamic versions of Flux Balance Analysis (FBA).
4. *Spatially resolved models.* These models focus on explicitly modeling the spatial distribution of microbial communities. Examples of this framework include Partial Differential Equations modeling motility and chemical diffusion.

In our modeling framework, we focus on exploring the impact that manipulating a subset of species (i.e., the driver species) has on the abundances of other species. To achieve that, a population dynamics model written as a set of ODEs is sufficient. More precisely, we make the following assumption:

Assumption 1.

a. The *state* of a microbial community can be determined by the abundance profile $x \in \mathbb{R}^N$ of its N species[1]. Here, the i-th entry x_i of this vector is the absolute abundance of the i-th species.
b. Spatial organization effects can be incorporated into the ecological network between the microbial species (e.g., determining if two species can interact or not).

[1]As in the main paper, here the term "species" is used in the general context of ecology, i.e., a set of organisms adapted to a particular set of resources in the environment. It doesn't necessarily represent the lowest major taxonomic rank. In fact, one could think of organizing microbes by strains, genera, or operational taxonomical units as well.

c. Host-microbe interactions and environmental factors remain constant during the time interval the control is performed.
d. Stochastic effects, such as drift [10, 11], are negligible.

We note that a more holistic assumption would consider that the state includes a combination of the abundance of the microbial species and the concentration of some biomolecules of the host. Assumption 1a can be relaxed in such way without changing the theoretical analysis that follows. Note also that, if necessary, the state of a microbial community could include the abundance of different strains of the same species. This can be useful if they have different effects to the host (e.g., in the case of metabolites produced by probiotic and pathogenic strains of *E. coli*). In analogy to the case of biochemical reactions, Assumption 1d is reasonable when the number of microbes of each species is sufficiently large and the microbial community is sufficiently "well-mixed".

Remark 1.

- Assumptions 1a and 1b justify the use of population dynamics model based on ODEs to describe the temporal evolution of the state of a microbial community.
- Due to Assumption 1c, host-microbe interactions and other environmental factors can be considered as constant parameters of such models.
- Assumption 1d allows us to use deterministic ODEs instead of stochastic ones, considerably simplifying the mathematical analysis.

We thus consider that the temporal evolution of the state $x(t) \in \mathbb{R}^N$ of the microbial community along time t can be described by the ODE model

$$\frac{\mathrm{d}x(t)}{\mathrm{d}t} := \dot{x}(t) = f(x(t)), \quad x(0) = x_0, \tag{S1}$$

where the vector field $f : \mathbb{R}^N \to \mathbb{R}^N$ models the population dynamics of the community, characterizing the intrinsic growth and intra/inter-species interactions. Here $x_0 \in \mathbb{R}^N$ represents the initial state of the community.

Examples of population dynamics of the above form are:

1. *The Generalized Lotka-Volterra (GLV) equations* with

$$f(x) = \mathsf{diag}(x)[Ax + r]. \tag{S2}$$

Here $A = (a_{ij}) \in \mathbb{R}^{N\times N}$ is the *interaction matrix*, $r \in \mathbb{R}^N$ is the *intrinsic growth rate* vector. The operator diag maps a vector $x \in \mathbb{R}^N$ into a diagonal matrix $\mathsf{diag}(x) \in \mathbb{R}^{N\times N}$ with diagonal entries $x_1, \cdots, x_N$. The GLV is the canonical population dynamic model for microbial communities in soils, lakes, human bodies, and even cheese [12–21].

2. *Pairwise interaction models*. In these models, the i-th entry $f_i(x)$ of $f(x)$ is given by

$$f_i(x) = q_i(x_i) + \sum_{j=1}^{N} a_{ij} h_{ij}(x_i, x_j), \quad i = 1, \cdots, N.$$

Here $A = (a_{ij}) \in \mathbb{R}^{N\times N}$ is the interaction matrix, $q_i : \mathbb{R} \to \mathbb{R}$ is the *intrinsic growth* of the i-th species, and $h_{ij} : \mathbb{R} \times \mathbb{R} \to \mathbb{R}$ is the so-called *functional response* of species j on species i. A standard and rather general form of the intrinsic growth is the logistic function [22]:

$$q_i(x_i) = x_i \left[\theta_{i,1} + \left(1 - \frac{x_i}{\theta_{i,2}}\right)\left(\frac{x_i}{\theta_{i,3}} - 1\right)\right], \tag{S3}$$

where the parameters $\theta_{i,1}, \theta_{i,2}$ and $\theta_{i,3}$ are the migration rate, the Allee constant and the carrying capacity for the i-th species, respectively. The so-called "Crowley-Martin" (CM) functional response is one of the most general ones, taking the form

$$h_{ij}(x_i, x_j) = \frac{\theta_{4,ij} x_i x_j}{(1 + \theta_{4,ij}\theta_{5,ij} x_i)(1 + \theta_{6,ij} x_j)}, \tag{S4}$$

for some constants parameters $\theta_{4,ij}, \theta_{5,ij}$ and $\theta_{6,ij}$ characterizing the saturation of the functional response. In predator-pray interactions, these three parameters model the capture rate, the handling time, and interference between predators, respectively. Note that the CM functional response includes as special cases the Lotka-Volterra, Holling-Type II and the DeAngelis-Beddington ones [22].

1.2. **Modeling control actions in microbial communities.** As described in the main paper, we consider the following control actions for controlling a microbial community:

1. *Prebiotics and bacteriostatic agents*. These are chemical compounds that modify the intrinsic growth rate of the actuated species. According to how the intrinsic growth rate is modeled in the GLV (S2), we model the effect of M such control actions as a continuous signal $u(t) \in \mathbb{R}^M, t \in \mathbb{R}$, modifying the dynamics of (S1) as follows:

$$\dot{x}(t) = f(x(t)) + g(x(t))u(t). \tag{S5}$$

Here $g(x) = [g_{ij}(x)] \in \mathbb{R}^{N\times M}$ is the *susceptibility matrix*, with g_{ij} determining the susceptibility of the i-th species to the j-th control action. At time t the j-th control action is a prebiotic if $u_j(t) > 0$, and a bacteriostatic agent if $u_j(t) < 0$.

A particular case of the above model is the controlled Generalized Lotka-Volterra model used in [15, 23], which takes the form

$$\dot{x}(t) = \mathsf{diag}(x(t))[Ax(t) + r + Bu(t)]. \tag{S6}$$

Here $A \in \mathbb{R}^{N\times N}$, $r \in \mathbb{R}^N$ and $B \in \mathbb{R}^{N\times M}$ are the interaction matrix, intrinsic growth rate vector and susceptibility matrix of the community, respectively. This model corresponds to $f(x) = \mathsf{diag}(x)[Ax + r]$ and $g(x) = \mathsf{diag}(x)B$. Note that the term $Bu(t)$ can be interpreted as modifying the intrinsic growth rates of the species.

2. *Transplantations and bactericides*. We model these control actions as instantaneously modifying the state of the microbial community, in the sense they instantaneously increase or decrease the abundance of the actuated species. Thus, we consider an impulsive control signal $u(t_k) \in \mathbb{R}^M$ applied at certain intervention times $\mathbb{T} = \{t_1, t_2, \cdots\}$, transforming the dynamics (S1) into the impulsive control system

$$\dot{x}(t) = f(x(t)), \quad \Delta x(t) = g(x(t))u(t). \tag{S7}$$

Above $g(x) = [g_{ij}(x)] \in \mathbb{R}^{N\times M}$ is again the susceptibility matrix and $\Delta x(t) := x(t^+) - x(t)$ denotes the *jump operator* at time t. Here $x(t^+) := \lim_{\tau \nearrow t} x(\tau)$. At the intervention instant $t_k \in \mathbb{T}$, the j-th control action is a transplantation if $u_j(t_k) > 0$, and a bactericide if $u_j(t_k) < 0$. We assume that $\mathbb{T}$ can be infinitely long and that it excludes the so-called "Zeno-behavior"[2].

In this case, the controlled GLV model used in the main text assumes constant susceptibilities, leading to the equation

$$\dot{x}(t) = \mathsf{diag}(x(t))[Ax(t) + r], \quad \Delta x(t) = Bu(t). \tag{S8}$$

Thus, control actions are classified as impulsive if they instantaneously modify the actuated species, and continuous otherwise.

1.3. **Controlled population dynamics with meromorphic functions.** As in the main text, we consider the following assumption:

Assumption 2. The pair $\{f(x), g(x)\}$ are *meromorphic functions* of x. Moreover, without loss of generality, we assume that $\mathsf{rank}\, g(x) = M$ for almost all $x \in \mathbb{R}^N$.

[2]That is, there is no accumulation point in $\mathbb{T}$. This means that there exists a constant $\tau_* > 0$ such that $t_{k+1} - t_k \geq \tau_*$ for all k.

A function is said *meromorphic* if it is the quotient of analytical functions [24]. Recall that analytic functions are those functions that admit Taylor series expansions. More generally, vector or matrix functions are meromorphic if each of their entries is meromorphic. Examples of analytical functions are polynomials and the trigonometric functions $\sin$ and $\cos$. Thus, both the logistic growth in Eq. (S3) and the Crowley-Martin functional response of Eq. (S4) are meromorphic functions. Assumption 2 is very mild in the sense that it is satisfied by most population dynamics models.

For $N = 1$ (i.e., $x \in \mathbb{R}$), an example of a function that is not meromorphic is

$$f(x) = \begin{cases} e^{-1/x^2} & \text{if } x \neq 0 \\ 0 & \text{if } x = 0 \end{cases}$$

Despite this function is infinitely differentiable (i.e., of class $\mathcal{C}^\infty$), this function is not meromorphic because it does not have the property of being generically zero nor generically non-zero. For further details we refer the reader to [24] and Supplementary Note 2.

2. Mathematical formalism to detect autonomous elements

2.1. **Notation.** We use rank to denote the rank of a constant matrix. The notation g-rank denotes the *generic rank* of a matrix with varying parameters. In what follows, all such matrices have meromorphic dependence on their parameters, so their generic rank is defined as the maximal rank that such matrix can attain when varying its parameters. Due to this fact, we emphasize that the generic rank is always larger or equal to the rank of the matrix at a given point of the parameter space [24]. The points at which the rank drops are called "singularities".

2.2. **Characterizing autonomous elements in continuous control systems.** For the continuous control systems of Eq. (S5) we have the following notions [24]:

Definition 1. An *autonomous element* of Eq. (S5) is a non-constant function $\xi(x)$ such that there exists an integer $\nu \geq 0$ and a meromorphic function F such that $F(\xi, \dot{\xi}, \cdots, \xi^{(\nu)}) = 0$.

Definition 2. System (S5) is said *accessible* if it has no autonomous element.

In the above setting, the chain rule provides a way to formally operate with one-forms, such as taking time derivatives: if $\omega = \beta^{\intercal}\mathrm{d}x$ then $\dot{\omega} := \dot{\beta}^{\intercal}\mathrm{d}x + \beta^{\intercal}\mathrm{d}\dot{x}$. To identify the presence of autonomous elements in the dynamics with continuous control of Eq. (S5), one calculates the sequence of subspaces $\mathcal{H}_k \subset \mathcal{X}$ defined recursively by

$$\mathcal{H}_k = \{\omega \in \mathcal{H}_k | \dot{\omega} \in \mathcal{H}_k\}, \quad k \geq 1, \tag{S9}$$

starting with $\mathcal{H}_1 = \mathcal{X}$. Then, we have the following result:

Theorem 1. [24, pp. 49, Thm. 3.17] System (S5) lacks autonomous elements if and only if there exists an integer $k^* \leq N + 1$ such that $\mathcal{H}_{k^*} = \{0\}$.

2.3. **Characterizing autonomous elements in impulsive control systems.** For the impulsive control systems of Eq. (S7), the notions of autonomous elements and accessibility are rather unexplored. Here we start by introducing a general definition of autonomous elements for this class of systems. Then, we provide a full characterization of their absence in a given controlled population dynamics.

First, note that an autonomous element is an internal variable of the system that is completely unaffected by the control actions. For the impulsive control systems of Eq. (S7), the control actions cause "jumps" in some state variables (i.e., discontinuities). Such jumps are then propagated to other state variables by the continuous dynamics. This observation motivates the following definition:

Definition 3. An *autonomous element* of (S7) is a non-constant function $\xi(x)$ such that $\xi(x(t)), t \in \mathbb{R}$, is a $\mathcal{C}^{\infty}$ function (i.e., infinitely differentiable function) under any impulsive input.

By analogy to the case with continuous control, we say that system (S7) is accessible if it has no autonomous element according to the above definition.

To characterize the accessibility of impulsive control systems, our strategy is to characterize the sequence of subspaces $\mathcal{H}_k$ of all functions of the state variables that can be differentiated at least $(k-1)$ times (hence these functions are "jump free"). The functions belonging to the limit $\mathcal{H}_\infty$ will be the autonomous elements of the system, since they are completely unaffected by the control actions. Consequently, if the limit subspace $\mathcal{H}_\infty$ is "integrable" (informally, it does not contain "fictitious" autonomous elements, see below for the mathematical definition), accessibility should be equivalent to the condition $\mathcal{H}_\infty = \{0\}$.

To implement the above strategy, we define a new "jump" operator Δ acting on a one-form $\omega = \beta^{\intercal}\mathrm{d}x \in \mathcal{X}$ as $\Delta\omega := \beta^{\intercal}\mathrm{d}\Delta x = \beta^{\intercal}\mathrm{d}\{g(x)u\}$. This new operator allows us to characterize the subspace $\mathcal{H}_2$ of $\mathcal{X}$ of all one-forms that do not have jumps as

$$\mathcal{H}_2 = \{\omega \in \mathcal{X} | \Delta\omega = 0\}. \tag{S10}$$

Next, the subspace of all one-form that can be differentiated once and do not have jumps is

$$\mathcal{H}_3 = \{\omega \in \mathcal{H}_2 | \ \dot{\omega} \text{ exists and } \dot{\omega} \in \mathcal{H}_2\}.$$

In general, for $k \geq 4$, the subspaces $\mathcal{H}_k$ of all one-forms that can be differentiated at least $k-1$ times and do not have jumps can be recursively calculated by

$$\mathcal{H}_k = \{\omega \in \mathcal{H}_{k-1} | \dot{\omega} \in \mathcal{H}_{k-1}\}, \quad k \geq 4.$$

Note that the subspace $\mathcal{H}_k$ characterizes all functions of the state that are $\mathcal{C}^{k-1}$-continuous. By construction, the above sequence is nested (i.e., $\mathcal{X} \supseteq \mathcal{H}_k \supseteq \mathcal{H}_{k+1}$) and each $\mathcal{H}_k$ is a finite-dimensional $\mathcal{K}$-vector space. Therefore, either the dimension decreases at least by one from $\mathcal{H}_k$ to $\mathcal{H}_{k+1}$, or otherwise it follows that $\mathcal{H}_{k+1} = \mathcal{H}_k$ for all subsequent k's. This last observation has the following implication: since the dimension of $\mathcal{H}_2$ is at most $N-M$ because $\mathsf{g\text{-}rank}\, g(x) = M$, then there exists $k^* \leq N - M$ such that $\mathcal{H}_{k^*} = \mathcal{H}_{k^*+1} = \cdots = \mathcal{H}_\infty$.

Consequently, since an autonomous element for impulsive control systems is identified with a $\mathcal{C}^\infty$ function, the accessibility of nonlinear impulsive systems has the following algebraic characterization:

Theorem 2. System (S7) is accessible if and only if $\mathcal{H}_\infty = \{0\}$.

Proof.

[Accessibility $\Rightarrow \mathcal{H}_\infty = \{0\}$] By contradiction. Suppose that there exists a non-zero one-form $\omega = \beta^\intercal \mathrm{d}x \in \mathcal{H}_\infty$. If this one-form is exact, $\omega = \mathrm{d}\varphi$ for some function φ, then $\varphi(x)$ is an autonomous element and the system is not accessible, concluding the proof of the claim. Hence we just need to show that $\mathcal{H}_\infty$ is always closed, i.e., it is spanned by a basis of exact one-forms. This comes from the fact that the subspaces $\mathcal{H}_k$, $k \geq 2$, coincide with the subspaces $\mathcal{H}_{k+1}$ defined for $\dot{x} = f(x) + g(x)u$ (cf. [24]), which are closed [25].

[$\mathcal{H}_\infty = \{0\} \Rightarrow$ Accessibility] By contradiction. If the system is not accessible, there exists non-constant $\xi(x)$ that is an autonomous element. Its associated one-form hence satisfies $\omega = \mathrm{d}\xi \in \mathcal{H}_2$. All its derivatives are also continuous, so $\omega \in \mathcal{H}_k$ for all k. Hence $\omega \in \mathcal{H}_\infty$, and $\mathcal{H}_\infty$ contains other elements in addition to the zero element. □

Remark 2. A consequence of Theorem 2 is that the accessibility properties of the impulsive control system of Eq. (S7) and the continuous control system of Eq. (S5) are identical. That is, the conditions for the absence of autonomous elements are identical in continuous and impulsive control systems. We note that this equivalence was only known for the linear systems $\dot{x} = Ax + Bu$ and $\dot{x} = Ax, \Delta x = Bu$ (see, e.g., [26] or [27]).

We also note that it is possible to construct in an unified way the subspaces $\mathcal{H}_k$ for continuous and impulsive control systems, using a mathematically more sophisticated framework based on the concept of "distributions" (also called "generalized functions"). For this, we denote by $\delta(t)$ the *Dirac delta* distribution at time t, which can be loosely defined as

$$\delta(t) = \begin{cases} +\infty & \text{if } t = 0 \\ 0 & \text{if } t \neq 0 \end{cases}$$

and satisfying the property $\int_{-\infty}^{\infty} \delta(t)\mathrm{d}t = 1$. [3] In the framework of distributions, the dynamics of the impulsive control system of Eq. (S7) can be rewritten as

$$\dot{x}(t) = f(x(t)) + g(x(t))u(t_k)\delta(t_k),$$

where $t_k \in \mathbb{T}$. Note that $\dot{x}(t) = f(x(t))$ if $t \neq t_k$. Given a one-form $\omega = \beta^\intercal \mathrm{d}x$ with $\beta \in \mathcal{K}^N$, we use the Chain Rule to define its time derivative $\dot{\omega}$ as:

- For $t \neq t_k$, we define $\dot{\omega} = \dot{\beta}^\intercal \mathrm{d}x + \beta^\intercal \mathrm{d}\dot{x} = \dot{\beta}^\intercal \mathrm{d}x + \beta^\intercal \mathrm{d}f$.
- For $t = t_k$, we define

$$\dot{\omega} = \dot{\beta}^\intercal \mathrm{d}x + \beta^\intercal \frac{\partial\{f(x) + g(x)u(t_k)\delta(t_k)\}}{\partial x}\mathrm{d}x + g(x)\delta(t_k)\mathrm{d}u.$$

[3] More formally, the Diract delta can be defined as a measure δ such that Lebesgue integral $\int_{-\infty}^{\infty} f(t)\delta\{\mathrm{d}t\} = f(0)$ for all continuous compactly supported functions f.

With a little abuse of notation, we denote by $\mathsf{span}_{\mathcal{K}}$ the span over meromorphic functions and distributions (i.e., impulses and more). Then, for impulsive control systems, the subspaces $\mathcal{H}_k$ can be defined as

$$\mathcal{H}_{k+1} = \{\omega \in \mathcal{H}_k | \dot{\omega} \in \mathcal{H}_k\}, \quad k \geq 1,$$

starting from $\mathcal{H}_1 = \mathsf{span}_{\mathcal{K}}\{\mathrm{d}x\}$. Note the above equation is identical to the characterization of the subspaces $\mathcal{H}_k$ for continuous control systems. Indeed, one can verify that $\mathcal{H}_2 = g(x)^{\perp}$, which is precisely Eq. (S10).

Example 1. Consider the microbial community with $N = 3$ species and $M = 1$ control inputs as in Fig. 1 of the main text. Its controlled population dynamics is given by

$$\begin{aligned} \dot{x}_1 &= 0.1 + x_1(1 - x_1/5)(x_1/3 - 1) - 0.1\frac{x_1 x_3}{1 + x_3}, \\ \dot{x}_2 &= 0.1 + x_2(1 - x_2/4)(x_2 - 1) + \frac{x_2 x_3}{1 + x_3}, \\ \dot{x}_3 &= x_3(1 - x_3/2)(x_3 - 1), \\ \Delta x_3 &= u. \end{aligned} \tag{S11}$$

Computing the sequence $\mathcal{H}_k$ one finds $\mathcal{H}_1 = \mathsf{span}_{\mathcal{K}}\{\mathrm{d}x_1, \mathrm{d}x_2, \mathrm{d}x_3\}, \mathcal{H}_2 = \mathsf{span}_{\mathcal{K}}\{\mathrm{d}x_2, \mathrm{d}x_1\}, \mathcal{H}_3 = \mathsf{span}_{\mathcal{K}}\{x_2\mathrm{d}x_1 + x_1\mathrm{d}x_2\}$ and $\mathcal{H}_4 = \{0\}$. Therefore, the controlled population dynamics is free of autonomous elements and hence accessible.

Example 2. For the microbial community of $N = 3$ species and $M = 1$ control inputs in Fig. 2 of the main text, its controlled population dynamics is given by the cGLV model

$$\begin{aligned} \dot{x}_1 &= x_1(-1 + x_3), \\ \dot{x}_2 &= x_2(1 - x_3), \\ \dot{x}_3 &= x_3(-0.5 + 1.5x_3) \\ \Delta x_3 &= u_1. \end{aligned} \tag{S12}$$

Computing the sequence $\mathcal{H}_k$ we find $\mathcal{H}_1 = \mathsf{span}_{\mathcal{K}}\{\mathrm{d}x_1, \mathrm{d}x_2, \mathrm{d}x_3\}$, $\mathcal{H}_2 = \mathsf{span}_{\mathcal{K}}\{\mathrm{d}x_1, \mathrm{d}x_2\}$ and $\mathcal{H}_k = \mathsf{span}_{\mathcal{K}}\{x_1\mathrm{d}x_2 + x_2\mathrm{d}x_1\}$ for $k \geq 3$. Indeed, for $\omega = x_1\mathrm{d}x_2 + x_2\mathrm{d}x_1$ one has that $\Delta\omega = 0$. Thus, since $\mathcal{H}_k \neq \{0\}$ for $k \geq 3$, the controlled population dynamics has an autonomous element. Note that the autonomous element can be obtained by integrating the one-form ω. Namely, since $\omega = \mathrm{d}(x_1 x_2)$, the autonomous element is $\xi = x_1 x_2$.

3. Detecting autonomous elements without knowledge of the population dynamics

3.1. **Characterizing the generic absence of autonomous elements.** We start by defining the graph (i.e., network) associated with a given pair of functions $\{f, g\}$ characterizing the controlled population dynamics of a microbial community:

Definition 4. Given a pair $\{f, g\}$ of meromorphic functions, its graph $\mathcal{G}_{f,g} = (\mathsf{X} \cup \mathsf{U}, \mathsf{E}_{f,g} \cup \mathsf{B}_{f,g})$ is the network with nodes $\mathsf{X} \cup \mathsf{U}$ corresponding to states and inputs, respectively, and edges $\mathsf{E}_{f,g} \cup \mathsf{B}_{f,g}$ determined by the following rules:

$$(\mathsf{x}_j \to \mathsf{x}_i) \in \mathsf{E}_{f,g} \Leftrightarrow J_{ij} \not\equiv 0, \quad (\mathsf{u}_j \to \mathsf{x}_i) \in \mathsf{B}_{f,g} \Leftrightarrow g_{ij} \not\equiv 0.$$

Above we denoted by $J_{ij}(x, u)$ and $g_{ij}(x)$ the (i, j)-th element of the matrices $J(x) = \partial\{f(x) + g(x)u\}/\partial x$ and $g(x)$, respectively. Thus the edges $(\mathsf{x}_j \to \mathsf{x}_i)$ and $(\mathsf{u}_j \to \mathsf{x}_i)$ simply mean that x_j and u_j appear in the right-hand side of the differential equation corresponding to $\dot{x}_i$, respectively.

Remark 3. For defining the graph $\mathcal{G}_{f,g}$ of a given pair $\{f, g\}$, it is instrumental to assume this pair is meromorphic. Only when this assumption is satisfied the conditions $J_{ij} \not\equiv 0$ and $g_{ij} \not\equiv 0$ have a well defining meaning (i.e., they mean these functions are identically zero).

Definition 5. Two meromorphic pairs $\{f, g\}$ and $\{\tilde{f}, \tilde{g}\}$ are *graph equivalent* if $\mathcal{G}_{\tilde{f},\tilde{g}} = \mathcal{G}_{f,g}$.

Note that $\{f, g\}$ is graph equivalent to itself, implying that graph-equivalence is an *equivalence relation*.

Given the controlled ecological network $\mathcal{G}^{\mathsf{C}}$ of the community, we characterize the class $\mathfrak{D}$ of all controlled population dynamics that the community may take as follows. The class $\mathfrak{D}$ contains all *base models* $\{f^*, g^*\}$ such that $\mathcal{G}_{f^*,g^*} = \mathcal{G}^{\mathsf{C}}$, together with all *deformations* of each of those base models. The base models, characterizing the simplest controlled dynamics that the community can take, are chosen as controlled GLV models with constant susceptibilities (see Eq.(4) of the main text). The notion of a deformation is defined as:

Definition 6. A meromorphic pair $\{f, g\}$ is a deformation of the base model $\{f^*, g^*\}$ if: (i) $\{f, g\}$ is graph equivalent to $\{f^*, g^*\}$; (ii) there exists a finite set of parameters $\theta \in \mathbb{R}^C$ such that $\{f(x), g(x)\} = \{\tilde{f}(x; \theta), \tilde{g}(x; \theta)\}$; and (iii) the identity $\{\tilde{f}(x; 0), \tilde{g}(x; 0)\} = \{f^*(x), g^*(x)\}$ holds.

For a given deformation $\{f, g\}$, its *size* is the minimum over all C for which conditions (i), (ii) and (iii) above are satisfied.

When we only know the ecological network underlying the microbial community and its base models, then the class $\mathfrak{D}$ characterizes all possible population dynamics that the microbial community can have. Because graph equivalence is an equivalence relation, the class $\mathfrak{D}$ is an equivalence class.

Definition 7. The class $\mathfrak{D}$ is said *structurally accessible* if almost all of its base models and almost all of their deformations lack autonomous elements.

Based on the above framework, our first result is the following:

Proposition 1. If $\{f^*, g^*\}$ lacks autonomous elements, then almost all of its deformations also lack autonomous elements.

Proof. Let $\{f, g\} \in \mathfrak{D}$ be a deformation. Since $\{f(x; 0), g(x; 0)\} = \{f^*(x), g^*(x)\}$ lacks autonomous elements, the meromorphic dependence of the function on their parameters imply that for almost all values of $\theta \in \mathbb{R}^C$ the corresponding deformation $\{f(x; \theta), g(x; \theta)\}$ lacks autonomous elements. □

The above proposition allows us to characterize the structural accessibility of $\mathfrak{D}$ by focusing only on the subclass $\mathfrak{D}_0 \subset \mathfrak{D}$ of deformations with size $C = 0$. That is, $\mathfrak{D}_0$ consists of all cGLV models $\{f^*, g^*\}$ such that $\mathcal{G}_{f^*,g^*} = \mathcal{G}^{\mathsf{C}}$. We next analyze the absence of autonomous elements in $\mathfrak{D}_0$.

3.2. **Generic absence of autonomous elements in cGLV models.** Recall that the base cGLV models $\{f^*, g^*\}$ constituting $\mathfrak{D}_0$ are given by

$$f^*(x) = \mathsf{diag}(x)[Ax + r], \quad g^*(x) = B.$$

By themselves, these models have three parameters (A, r, B), corresponding to the interaction matrix, intrinsic growth vector, and susceptibility matrix of the community, respectively. By assumption, we consider that $\mathsf{rank}\, B = M$. Thus, the subclass $\mathfrak{D}_0$ is parametrized by the set of all triplets (A, r, B) such that the graph $\mathcal{G}_{f^*,g^*}$ coincides with $\mathcal{G}^{\mathsf{C}}$.

The next result characterizes the lack of autonomous elements in almost all the base models in $\mathfrak{D}_0$.

Proposition 2. Almost all cGLV models lack autonomous elements iff (i) each species node can be reached by a path that starts in a control-input node, and (ii) $\mathsf{g\text{-}rank}\begin{pmatrix} A & B \end{pmatrix} = N$.

Proof. Since the absence of autonomous elements guarantees that the system can explore a full N-dimensional space, we assume that $x_i > 0$ without loss of generality. Additionally, since the accessibility properties with continuous and impulsive control are identical (see Remark 2), we consider without loss of generality the cGLV models with impulsive control. In coordinates $z = \ln(x)$ (i.e., $x = e^z$ interpreted component-wise), the dynamics cGLV dynamics can be written as

$$\dot{z} = r + Ae^z, \quad \Delta z = Bv, \tag{S13}$$

where $v = B^+ [\ln(e^z + Bu) - z]$ is a new input. Here B^+ denotes the Moore-Penrose pseudoinverse of B that, since B has full column rank, satisfies $B^+B = I$ (i.e., $u = B^+[e^{z+Bv} - x]$).

($\Rightarrow$) By contradiction, showing that not satisfying condition (i) or (ii) implies the existence of an autonomous element. If condition (i) is not satisfied, the state variables associated to those nodes are autonomous elements. If condition (ii) is not satisfied then $\mathsf{g\text{-}rank}\begin{pmatrix} A & B \end{pmatrix} < N$. In turn, this condition implies that $\mathsf{rank}\begin{pmatrix} A & B \end{pmatrix} < N$. Therefore there exists $\beta \in \mathbb{R}^N$ such that $\beta^\intercal A = \beta^\intercal B = 0$. Consequently $\xi = \beta^\intercal z$ is an autonomous element because $\dot{\xi} = \beta^\intercal r$ and $\Delta\xi = 0$.

($\Leftarrow$) Under conditions (i) and (ii), the pair (A, B) is structurally controllable. This means that almost all pairs (A, B) are linearly controllable. Next we consider the linearization (S13) at $z = 0$ which coincides with the linear system $\dot{\delta}_z = A\delta_z$, $\Delta\delta_z = Bv$. Due to the linear structural controllability, for almost all pairs (A, B) this linearization is controllable. The claim follows since linear controllability is a sufficient condition for the absence of autonomous elements in the original nonlinear system.

□

Remark 4.

1. Proposition 2 shows that the growth rate vector r does not play any role in the generic presence/absence of autonomous elements in the cGLV model.
2. Note that for cGLV models, a self-loop in their graph could exists due to intrinsic growth terms $r_i x_i$, or due to intra-species interactions $a_{ii} x_i x_j$.

To obtain a graph-characterization of the structural accessibility of the class $\mathfrak{D}$, it will be useful to recall the following result:

Lemma 1. (Lemma 2 in [28]) The condition $\mathsf{g\text{-}rank}\begin{pmatrix} A & B \end{pmatrix} = N$ admits the following equivalent graph characterization: there exists a disjoint union of paths starting at the input nodes and cycles that cover all state nodes.

As discussed in Remark 4.2, given the ecological network $\mathcal{G}^{\mathsf{C}}$ of a microbial community, we cannot know if self-loops occur due to the presence of intrinsic growth terms or due to intra-species interactions. For example, if the only incoming edge to species x_i is the self-loop $\mathsf{x}_i \to \mathsf{x}_i$, this could correspond to three different dynamics:

$$\dot{x}_i = r_i x_i \quad \text{or} \quad \dot{x}_i = r_i x_i + a_{ii} x_i^2 \quad \text{or} \quad \dot{x}_i = a_{ii} x_i^2.$$

Considering Remark 4.1, the intrinsic growth rates do not play any role in the absence or presence of autonomous elements in the cGLV model. By contrast, intra-species interactions can contribute to remove autonomous elements by helping the pair (A, B) to be linearly structurally controllable. Therefore, being agnostic to whether a self-loop corresponds to an intrinsic growth term or an intra-species interaction (or both), we should consider as worst case that all self-loops in $\mathcal{G}^{\mathsf{C}}$ occur due to intrinsic growth terms. With this observation, we obtain the following graph-characterization of structural accessibility:

Theorem 3. The class $\mathfrak{D}$ is structurally accessible if and only if: (i) each state node is the end-node of a U-rooted path, and (ii) there is a disjoint union of paths that start in input nodes and cycles (excluding self-loops) that cover state nodes.

Proof. Proposition 2 and Lemma 1 imply that almost all cGLV models in $\mathfrak{D}_0$ lack autonomous elements if and only if conditions (i) and (ii) are satisfied. Then, the claim follows by using Proposition 1. □

4. Identifying a minimal set of driver species

Let $\mathcal{G} = (\mathsf{X}, \mathsf{E})$ denote the ecological network underlying the microbial community. To simplify the notation, recall we also denote this graph as $\mathcal{G}(\mathsf{X})$ or simply $\mathcal{G}$. Note that $\mathcal{G}$ is a directed graph or digraph. Next consider the controlled ecological network $\mathcal{G}^{\mathsf{C}} = (\mathsf{X} \cup \mathsf{U}, \mathsf{E} \cup \mathsf{B})$ obtained by adding M control input nodes $\mathsf{U} = \{\mathsf{u}_1, \cdots, \mathsf{u}_M\}$ and edges of form $(\mathsf{u}_j \to \mathsf{x}_i) \in \mathsf{B}$. Recall that the control input u_j is said to actuate species x_i iff $(\mathsf{u}_j \to \mathsf{x}_i) \in \mathsf{B}$.

We will use of the following notions:

Definition 8.

a) The controlled network $\mathcal{G}^{\mathsf{C}}$ is said to have a *dedicated input configuration* if each control input actuates a single species.

b) $\mathcal{G}^{\mathsf{C}}$ is said to have a *feasible* dedicated input configuration if it has a dedicated input configuration and it is structurally accessible. If in addition $\mathcal{G}^{\mathsf{C}}$ has the minimal possible number of control input nodes, then $\mathcal{G}^{\mathsf{C}}$ is said to have a *minimal* feasible dedicated input configuration (mFDIC).

The notion of a "dedicated input configuration" was introduced in Ref. [29] in the context of (linear) structural controllability for linear systems. Definition 8 above is an extension to nonlinear systems in the context of the notion of structural accessibility.

Note that if $\mathcal{G}^{\mathsf{C}}$ has a mFDIC, then the set of actuated species $\mathsf{X}_D \subseteq \mathsf{X}$ in $\mathcal{G}^{\mathsf{C}}$ is a minimal set of driver species. Conversely, if $\mathsf{X}_D \subseteq \mathsf{X}$ is a minimal set of driver species, then we can build a mFDIC just by actuating each driver species with a different control input. As a consequence, a minimal set of driver species can be characterized as a mFDIC. Using this observation, we can identify a minimal set of driver species by using the methods to find mFDICs developed in Ref. [29].

More precisely, let $\tilde{\mathcal{G}}(\mathsf{X})$ be the subgraph obtained by removing all self-loops in $\mathcal{G}(\mathsf{X})$. Let $\tilde{\mathcal{B}}(\mathsf{X}^- \cup \mathsf{X}^+)$ be the bipartite representation of $\tilde{\mathcal{G}}(\mathsf{X})$, built by placing the edge $(\mathsf{x}_j^+, \mathsf{x}_i^-)$ in $\tilde{\mathcal{B}}$ if there is a directed edge $(\mathsf{x}_j \to \mathsf{x}_i)$ in $\tilde{\mathcal{G}}$. Next, let us introduce the following notions:

Definition 9. [29]

a) A strongly connected component (SCC) is called *non-top linked* if it has no incoming edges from other SCCs.

b) Consider the bipartite representation $\tilde{\mathcal{B}}(\mathsf{X}^- \cup \mathsf{X}^+)$ of $\tilde{\mathcal{G}}(\mathsf{X})$. Let M^* be a maximum matching in $\tilde{\mathcal{B}}$. Then, a non-top linked SCC is said to be *top assignable* with respect to M^* if it contains at least one right-unmatched node in M^*.

Finally, let $\mathsf{Z} \subseteq \mathsf{X}$ be the set of right-unmatched nodes of some maximum matching of $\tilde{\mathcal{B}}$ with maximum top assignability. Let $\mathsf{W} \subseteq \mathsf{X}$ be a set consisting of one state node from each non-top linked SCC of $\tilde{\mathcal{G}}$ not already present in Z. Then we have the following result:

Proposition 3. A set $\mathsf{X}_D \subseteq \mathsf{X}$ is a minimal set of driver species if and only if there exist two disjoint subsets Z and W, as defined above, such that $\mathsf{X}_D = \mathsf{Z} \cup \mathsf{W}$.

Proof. Under the assumptions of Proposition 3, Theorem 5 of [29] implies that the resulting $\tilde{\mathcal{G}}^{\mathsf{C}}$ is linearly structurally controllable. Then, Theorem 1 of [30] implies that $\tilde{\mathcal{G}}^{\mathsf{C}}$ satisfies the following two conditions: (i) each state node is the end-node of a U-rooted path, and (ii) there is a disjoint union of paths that start in input nodes and cycles that cover all state nodes. Finally, by recalling that $\tilde{\mathcal{G}}$ was obtained from $\mathcal{G}$ by discarding all self-loops, it follows from Theorem 3 that $\mathcal{G}^{\mathsf{C}}$ is structurally accessible. □

Using the above Proposition, we can identify a minimal set of driver species by applying Algorithm 1 of [29] to $\tilde{\mathcal{G}}$. We implemented this algorithm in Julia as the `DriverSpecies` function in the `DriverSpeciesModule` package, available at the following GitHub repository:

`https://github.com/mtangulo/DriverSpecies`

Remark 5. From Theorem 11 of [29], it follows that the algorithm to find a minimal set of driver species has complexity $O(N^3)$. For communities with $N = 100$ species, the provided Julia implementation finds a minimal set of driver species in milliseconds.

5. Sufficient number of impulses to drive a microbial community

We start recalling the following elementary notions [24]:

Definition 10. A one-form $\omega \in \mathcal{X}$ is closed if $\mathrm{d}\omega = 0$.

Examples of closed one-forms are exact one-forms, i.e., ω's for which there exists a meromorphic function $\phi \in \mathcal{K}$ such that $\mathrm{d}\phi = \omega$.

Consider now a given pair $\{f, g\}$ and its associated sequence of subspaces $\{\mathcal{H}_k, k \geq 1\}$.

Definition 11. A subspace $\mathcal{H}_k \subseteq \mathcal{X}$ is closed if it has a basis of closed one-forms.

We then have the following characterization:

Theorem 4. Consider the impulsive control system of Eq. (S7). Assume that there exists integer k^* such that $\mathcal{H}_{k^*} = \{0\}$ and all subspaces $\mathcal{H}_k$'s, $k \leq k^*$ are closed. Then $L = k^* - 1$ impulses suffice to locally steer the system to almost any desired state.

Proof. Under these assumptions, the static feedback linearization theorem [24, Theorem 9.1] ensures that there exists local coordinates $\zeta = \varphi(x)$ and inputs $v \in \mathbb{R}^M$ (i.e., $u = g^+(x)v$) such that the system reads as

$$\zeta_i^{(r_i)} = \alpha_i(x), \quad \Delta\zeta_i = v_i; \quad i = 1, \cdots M.$$

Here, $r_i \geq 1$ are some integers and and $\alpha_i(x)$ is some nonlinear functions. To complete the proof of the theorem, we first show that the above system can be steered to any desired state using r_i impulses at times $\mathbb{T} = \{t_0, t_1, \cdots, t_{r_i - 1}\}$. Finally, since the integers r_i's are the so-called controllability indices of the system and they satisfy $k^* - 1 = \max\{r_1, \cdots, r_M\}$ (see e.g., [24, Section 3.7]), the claim follows.

Define the vector $z_i \in \mathbb{R}^{r_i}$ as $z_{i,1} = \zeta_i$ and $z_{i,k} = \zeta_i^{(k-1)}$ (i.e., $k-1$ time derivative). Then, the above dynamics can be rewritten as

$$\dot{z}_i(t) = A_0 z_i(t) + b\alpha_i(t), \quad \Delta z_i(t) = b v_i(t), \quad i = 1, \cdots, M, \tag{S14}$$

where $\alpha_i(t) := \alpha_i(x(t))$, and

$$A_0 = \begin{pmatrix} 0 & 1 & 0 & \cdots & 0 \\ 0 & 0 & 1 & \cdots & 0 \\ \vdots & & & \ddots & \\ 0 & & 0 & \cdots & 1 \\ 0 & 0 & 0 & \cdots & 0 \end{pmatrix}, \quad b = \begin{pmatrix} 0 \\ 0 \\ \vdots \\ 0 \\ 1 \end{pmatrix}.$$

Consider an initial state $z_i(t_0)$. Then:

- The impulse $v_i(t_0)$ at t_0 will set $z_i(t_0^+) = z_i(t_0) + b v_i(t_0)$.
- Using the variation of parameters formula, flowing from t_0 to t_1 yields

$$z_i(t_1) = e^{A_0 t_1}[z_i(t_0) + b v_i(t_0)] + \int_{t_0}^{t_1} e^{A_0(t_1 - \tau)} b\alpha(\tau)\mathrm{d}\tau.$$

- The impulse $v_i(t_1)$ at t_1 will set

$$z_i(t_1^+) = b v_i(t_1) + e^{A_0 t_1} b v_i(t_0) + \gamma_1,$$

  where $\gamma_1 = e^{A_0 t_1} z_i(t_0) + \int_{t_0}^{t_1} e^{A_0(t_1-\tau)} b\alpha(\tau)\mathrm{d}\tau$.
- Flowing from t_1 to t_2 yields

$$z_i(t_2) = e^{A_0 t_2} b v_i(t_1) + e^{A_0(t_1+t_2)} b v_i(t_0) + \gamma_2$$

  where $\gamma_2 = e^{A_0(t_1+t_2)} z_i(t_0) + e^{A_0 t_2} \int_{t_0}^{t_1} e^{A_0(t_1-\tau)} b\alpha(\tau)\mathrm{d}\tau + \int_{t_1}^{t_2} e^{A(t_2-\tau)} b\alpha(\tau)\mathrm{d}\tau$. Here we have used the identity $e^{A_0 t_1} e^{A_0 t_2} = e^{A(t_1+t_2)}$.

- The impulse $v_i(t_2)$ at t_2 will set

$$z_i(t_2^+) = bv_i(t_2) + e^{A_0 t_2} bv_i(t_1) + e^{A_0(t_1+t_2)} bv_i(t_0) + \gamma_2.$$

Repeating the above process $r_i - 1$ times, we obtain the expression

$$z_i(t_{r_i-1}^+) = CV_i + \Gamma,$$

where the matrix $C \in \mathbb{R}^{r_i \times r_i}$ is given[4] by

$$C = \begin{pmatrix} b & e^{A_0 T_{r_i-1}} b & e^{A_0 T_{r_i-2}} b & \cdots & e^{A_0 T_1} b \end{pmatrix},$$

with $T_{r_i-1} = \sum_{k=r_i-1}^{1} t_k$. That is $T_{r_i-1} = t_{r_i-1}$, $T_{r_i-2} = t_{r_i-1} + t_{r_i-2}$, ..., $T_1 = t_{r_i-1} + \cdots + t_1 + t_0$. The vector $V_i \in \mathbb{R}^{r_i}$ contains all impulses:

$$V_i = \begin{pmatrix} v_i(t_{r_i-1}) & v_i(t_{r_i-2}) & \cdots & v_i(t_1) & v_i(t_0) \end{pmatrix}^{\mathsf{T}},$$

and $\Gamma \in \mathbb{R}^{r_i}$ is the vector of the scalars γ_i.

Due to the form of A_0, direct computation shows that

$$e^{A_0 T_k} b = \begin{pmatrix} \frac{T_k^{r_i-1}}{(r_i-1)!} & \frac{T_k^{r_i-2}}{(r_i-2)!} & \cdots & \frac{T_k^2}{2!} & T_k & 1 \end{pmatrix}^{\mathsf{T}}.$$

Since all t_k's are different by assumption, this implies that all columns of C are linearly independent and thus $\operatorname{rank} C = r_i$. Therefore, choosing the j-th control impulse as $u(t_j) = V_{ij}*$ with $V_i^* = C^{-1}(z_i^* - \Gamma)$ ensures that $z_i(t_{r_i-1}^+) = z_i^*$ as desired. □

Remark 6. The case when $\mathcal{H}_{k^*} = \{0\}$ for some k^*, but some $\mathcal{H}_k$ is not closed, suggests that the system can only be locally asymptotically steered as $t \to \infty$.

Example 3. Consider a microbial community with $N = 3$ species, $M = 1$ control input, and the following controlled population dynamics:

$$\dot{x}_1 = \frac{1 - x_1 + x_3}{1 + x_3}, \quad \dot{x}_2 = \frac{1 - x_3}{1 + x_3}, \quad \dot{x}_3 = 0, \quad \Delta x_3 = u.$$

Computing the sequence $\mathcal{H}_k$ one finds:

$$\mathcal{H}_1 = \operatorname*{span}_{\mathcal{K}}\{\mathrm{d}x_1, \mathrm{d}x_2, \mathrm{d}x_3\}, \quad \mathcal{H}_2 = \operatorname*{span}_{\mathcal{K}}\{\mathrm{d}x_2, \mathrm{d}x_1\}, \quad \mathcal{H}_3 = \operatorname*{span}_{\mathcal{K}}\{2\mathrm{d}x_1 + x_1\mathrm{d}x_2\}, \quad \mathcal{H}_4 = \{0\}$$

implying that the system is free of autonomous elements and hence accessible. To apply Theorem 4, note that $\mathcal{H}_1$ and $\mathcal{H}_2$ are obviously integrable. The subspace $\mathcal{H}_3$ is also integrable, since $\omega = 2\mathrm{d}x_1 + x_1\mathrm{d}x_2$ can be integrated to $\mathrm{d}(e^{x_1}x_2/2)$. Therefore, $L = 3$ impulses are required to steer this microbial community.

We also use this example to illustrate the procedure that leads to Eq. (S14). In this example, using the coordinates

$$z_1 = e^{x_2/2}x_1, \quad z_2 = -\frac{e^{x_2/2}}{2}(-2 + x_1), \quad z_3 = \frac{e^{x_2/2}}{4(1 + x_3)}(-4x_3 + x_1 + x_1x_3)$$

the system reads as

$$\dot{z}_1 = z_2, \quad \dot{z}_2 = z_3, \quad \dot{z}_3 = \alpha(z),$$

where $\alpha(z) = (z_2^2 - z_1z_2 + 2z_2z_3 + 4z_3^2)/(2z_1 + 4z_2)$. In addition, since $x_3 = (z_1 - 4z_3)/(z_2 + 4(z_2 + z_3))$, choosing

$$u = \frac{z_1 - 4(z_3 + v)}{z_2 + 4(z_2 + z_3 + v)} - \frac{z_1 - 4z_3}{z_2 + 4(z_2 + z_3)},$$

where $v \in \mathbb{R}$ is a new control input, leads to the equation

$$\Delta z_3 = v.$$

[4]Note here C is a matrix, not the size of a deformation.

Example 4. For the microbial community of Fig. 1 in the main text, we previously calculated that

$$\mathcal{H}_1 = \underset{\mathcal{K}}{\mathrm{span}}\{\mathrm{d}x_1, \mathrm{d}x_2, \mathrm{d}x_3\}, \quad \mathcal{H}_2 = \underset{\mathcal{K}}{\mathrm{span}}\{\mathrm{d}x_2, \mathrm{d}x_1\}, \quad \mathcal{H}_3 = \underset{\mathcal{K}}{\mathrm{span}}\{x_2\mathrm{d}x_1 + x_1\mathrm{d}x_2\}, \quad \mathcal{H}_4 = \{0\}.$$

The subspaces $\mathcal{H}_1$ and $\mathcal{H}_2$ are obviously closed. The subspace $\mathcal{H}_3$ is closed because $x_2\mathrm{d}x_1 + x_1\mathrm{d}x_2 = \mathrm{d}(x_1x_2)$ and $\mathrm{d}(\mathrm{d}x_1x_2) = 0$. Therefore, an horizon of $L = 3$ control actions can steer this microbial community to the desired state.

Remark 7. Some remarks are in order:

a. In general, the condition that the subspaces $\mathcal{H}_k$ are closed does not depend on the "complexity" of the dynamics in the sense of how complicated are its defining equations. Indeed, even the base cGLV dynamics can produce non-closed subspaces.
b. The key step in the proof of Theorem 4 is the transformation $\varphi(x)$ into the so-called "cascade form" of Eq. (S14). This allows us to calculate the solution of the system trajectories and to calculate the impulsive control inputs that will steer the system towards the desired state. Indeed, this same method can be used to calculate the control inputs even if the system is not linearized. The only requirement is that the graph of the system is acyclic.

Example 5. To illustrate Remark 7a, consider the following cGLV with one control input:

$$\begin{aligned} \dot{x}_1 &= x_1(x_1 + x_2) \\ \dot{x}_2 &= x_2(x_1 + 2x_2) \\ \dot{x}_3 &= x_3(x_1 + 2x_2 + 3x_3) \\ \Delta x_1 &= \Delta x_2 = \Delta x_3 = u. \end{aligned}$$

One can compute

$$\mathcal{H}_1 = \underset{\mathcal{K}}{\mathrm{span}}\{\mathrm{d}x_1, \mathrm{d}x_2, \mathrm{d}x_3\}, \quad \mathcal{H}_2 = \underset{\mathcal{K}}{\mathrm{span}}\{-\mathrm{d}x_1 + \mathrm{d}x_3, -\mathrm{d}x_1 + \mathrm{d}x_3\},$$

$$\mathcal{H}_3 = \underset{\mathcal{K}}{\mathrm{span}}\{3(x_2 - 3x_3)\mathrm{d}x_1 + (-2x_1 + x_2 + 9x_3)\mathrm{d}x_2 + 2(x_1 - 2x_2)\mathrm{d}x_3\}, \quad \mathcal{H}_4 = \{0\}.$$

Here $\mathcal{H}_3$ is not closed.

Example 6. To illustrate Remark 7b, we consider the microbial community in Fig. 2b of the main text. This community has the cGLV controlled population dynamics

$$\begin{aligned} \dot{x}_1 &= x_1(-1 + x_3) \\ \dot{x}_2 &= x_2(1 - x_3) \\ \dot{x}_3 &= x_3(-0.5 + 1.5x_3) \\ \Delta x_1 &= u_2 \\ \Delta x_3 &= u_1. \end{aligned}$$

Denote the initial state as $x(0) = (x_{10}, x_{20}, x_{30})^\intercal$, and the desired state as $x_d = (x_{1d}, x_{2d}, x_{3d})^\intercal$.

Let us choose $t_0 = 0$ as the first intervention instant and define $u_1(t_0) = u_{10}$. Considering the third equation $\dot{x}_3 = x_3(-0.5 + 1.5x_3)$ with the initial condition $x_{30} + u_{10}$ obtained after the first intervention, its solution at time t can be calculated by direct integration as

$$x_3(t) = \frac{1.}{\frac{e^{0.5t}(1-3(u_{10}+x_{30}))}{u_{10}+x_{30}} + 3}.$$

Consider now the second equation $\dot{x}_2 = x_2(1 - x_3)$. Using the above expression for $x_3(t)$, the abundance of x_2 at the time of the second intervention t_1 can be again obtained by direct integration:

$$\int_{x_{20}}^{x_2(t_1)} \frac{\mathrm{d}x_2}{x_2} = \int_0^{t_1} [1 - x_3(t)]\mathrm{d}t,$$

leading to

$$\ln x_2(t_1) - \ln x_{20} = \frac{2}{3}\ln\left(u_{10}\left(1 - e^{0.5t_1}\right) + x_{30}\left(1 - e^{0.5t_1}\right) + \frac{1}{3}e^{0.5t_1}\right) + \frac{2}{3}t_1 + 0.732408.$$

Choosing the value u_{10} such that $x_2(t_1) = x_{2d}$ we obtain

$$u_{10} = -\frac{-\frac{\frac{1}{3}x_{2d}^{1.5}}{x_{20}^{1.5}} + \left(e^{t_1} - e^{1.5t_1}\right)x_{30} + \frac{1}{3}e^{1.5t_1}}{e^{t_1} - e^{1.5t_1}}. \tag{S15}$$

Therefore, choosing the control actions as

$$u(t_0) = (u_{10}, 0)^\intercal, \quad u(t_1) = (x_{3d} - x_2(t_1), x_{1d} - x_1(t_1))^\intercal, \tag{S16}$$

guarantee that $x(t_1) = x_d$, as desired.

Remark 8. Note that in the extreme case of driving all N species we obtain g-rank $g(x) = N$, implying that $\mathcal{H}_2 = \{0\}$ which is evidently exact. Indeed, in this case a single impulse is sufficient to steer the system to almost any desired state. This fact suggests that, by increasing the number of control inputs (i.e., driver species), it may be possible to drive the system to the desired state using a finite number of impulses.

Remark 9. There exists a tradeoff between the number of driver species and the complexity of the control signal needed to drive the microbial community towards the desired state. To illustrate this point, let's consider the microbial community of Example 6 with $N = 3$ species and $M = 2$ driver species. Here it is necessary to use the two impulsive control inputs of Eq. (S16) to reach the desired state. The component u_{10} of these control inputs is rather complicated as shown in Eq. (S15). By contrast, if we enlarge the set of driver species to $M = 3$ (i.e., we control all species), then a single control input $u(t_0) = x_d - x(t)$ can evidently drive the microbial community to x_d. Note also that the control signal is simpler compared to the case of using $M = 2$ driver species. In general, it can be expected that the complexity of the signal needed to drive the community increases as the number of driver species decreases.

6. Calculating control inputs for microbial communities with uncertain population dynamics or large number of species

We consider the linear pair $\{\hat{A}x, \hat{B}\}$ to predict the states $\hat{X}_{k,L} = \{\hat{x}(t_{k+1}), \cdots, \hat{x}(t_{k+L+1})\}$ that the community will take in response to a sequence of L impulsive control inputs $U_{k,L} = \{u(t_k), \cdots, u(t_{k+L-1})\}$. For simplicity, we assume that $t_{k+1} - t_k = \tau$ for all k.

As discussed in the main text, at each intervention instant $t_k \in \mathbb{T}$, the control input is chosen as $u(t_k) = u_1^*(t_k)$. Here $u_1^*(t_k)$ is the first element of the optimal control input sequence $U_{k,L}^*$ calculated by solving the optimization problem

$$U_{k,L}^* = \arg \min_{U_{k,L} \in \mathbb{R}^{M \times L}} J_{x_\mathrm{d}}(\hat{X}_{k,L}, U_{k,L}), \tag{S17}$$

where the cost function J_{x_d} is quadratic:

$$J_{x_\mathrm{d}}(\hat{X}_{k,L}, U_{k,L}) = \sum_{i=k}^{L} [\hat{x}(t_i) - x_\mathrm{d}]^\intercal Q [\hat{x}(t_i) - x_\mathrm{d}] + u(t_i)^\intercal R u(t_i). \tag{S18}$$

The matrices $Q = Q^\intercal \in \mathbb{R}^{N \times N}, Q \succ 0$, and $R = R^\intercal \in \mathbb{R}^{M \times M}$, $R \succ 0$, penalize deviations from the desired state and control "effort", respectively. In this cost function, we choose the matrix Q such that $(Q, e^{\hat{A}\tau})$ is observable.

Note that using the linear pair $\{\hat{A}x, \hat{B}\}$ the predicted state of the community satisfies

$$\dot{\hat{x}}(t) = \hat{A}x(t) \text{ if } t \notin \mathbb{T}; \quad \Delta\hat{x}(t) = \hat{B}u(t) \text{ if } t \in \mathbb{T}.$$

Therefore, we can compute the predicted state $\hat{x}(t_{k+1})$ that the community will take after we apply the control input $u(t_k)$ by integrating the above equation between the intervention instants t_{k+1} and t_k. This yields the recursive discrete-time equations

$$\hat{x}(t_{k+1}) = e^{\hat{A}\tau}\hat{x}(t_k) + e^{\hat{A}\tau}\hat{B}u(t_k). \tag{S19}$$

With this observation, solving the optimization problem of Eq. (S17) becomes equivalent to minimizing the cost function of Eq. (S18) subject to the linear discrete-time dynamics (S19). The solution to this minimization problem is a well-known Linear Quadratic Regulator, see e.g., [31]. In the particular case of an infinite prediction horizon $L \to \infty$, this implies that the solution to the optimization problem of Eq. (S17) takes the form of the linear feedback controller $u(t_k) = K(\hat{x}(t_k) - x_\mathrm{d})$ where $K \in \mathbb{R}^{M \times N}$ is the gain matrix. Since we can measure the current state of the community, the predicted value of the current state $\hat{x}(t_k)$ simply coincides with the current state $x(t_k)$, giving the final form for the controller:

$$u(t_k) = K(x(t_k) - x_\mathrm{d}). \tag{S20}$$

Above, the gain matrix is given by $K = [(e^{\hat{A}\tau}\hat{B})^\intercal P e^{\hat{A}\tau}\hat{B}]^{-1}[(e^{\hat{A}\tau}\hat{B})^\intercal P e^{\hat{A}\tau}]$ with $P \in \mathbb{R}^{N \times N}$ the solution to the Riccati equation

$$e^{\hat{A}^\intercal \tau} P e^{\hat{A}\tau} - P - [e^{\hat{A}^\intercal \tau} P e^{\hat{A}\tau}\hat{B}](B^\intercal P e^{\hat{A}\tau}\hat{B})^{-1}(e^{\hat{A}\tau}\hat{B})^\intercal P e^{\hat{A}\tau} + Q = 0.$$

For the results in the main paper, the matrix gain K was computed using the Julia command `dlqr` in the `ControlSystems` module.

Remark 10. In the case of continuous control, the solution to the analogous optimization problem

$$\arg\min \int_{t=0}^{\infty} [\hat{x}(t) - x_\mathrm{d}]^\intercal Q [\hat{x}(t) - x_\mathrm{d}] + u(t)^\intercal R u(t) \mathrm{d}t,$$

subject to $\dot{\hat{x}}(t) = \hat{A}x(t) + \hat{B}u(t)$ also takes the form of the linear feedback controller $u(t) = K(\hat{x}(t) - x_\mathrm{d}) = K(x(t) - x_\mathrm{d})$, see, e.g., [32, pp. 194] or [31,33]. In this case, the gain-matrix is $K = R^{-1}B^\intercal P$ with P the solution to the Riccati equation

$$\hat{A}^\intercal P + P\hat{A} + P\hat{B}R^{-1}\hat{B}^\intercal P + Q = 0.$$

Remark 11. The performance of the linear MPC also depends on the chosen $(\hat{A}, \hat{B})$ and the desired state. In general, the linear MPC is guaranteed to succeed only if the desired state is "close enough" to the initial state. In this statement, how "close" or "far" is a desired state depends on how well the linear dynamics approximates the true population dynamics of the community. If the approximation is perfect, any desired state in $\mathbb{R}^N$ can be reached using a linear MPC with arbitrary $Q \succ 0$ and $R \succ 0$.

Remark 12. Since the linear impulsive and continuous MPCs we proposed here coincide with the solution to discrete and continuous LQR problems, respectively, our proposed controllers will naturally inherit the robustness properties of LQRs. The excellent robustness properties of continuous time LQRs (such as 50% gain reduction tolerance, $+\infty$ gain margin, and 60 degrees of phase margin) were derived in the 70's in a series of now classical papers by Safonov, Athans, Rosenbrock and others, summarized in books such as [33]. Similarly, the robustness properties of discrete time LQRs were derived in seminal contributions from the 80's, such as [34].

Remark 13. In Ref. [35], the authors proposed a strategy to optimally control complex networks based on the linearization of their nonlinear dynamics. To apply this approach for controlling microbial communities, it would be necessary to have exact knowledge of their population dynamics in order to calculate the linearization. Our approach circumvents this limitation, requiring only to know a proxy $(\hat{A}, \hat{B})$ of the interaction matrix and susceptibility matrix of the community. We emphasize that $(\hat{A}, \hat{B})$ does not need to coincide with the linearization of the dynamics (see, e.g., Fig. 4c,d and Fig. 5 of the main text).

7. Simulation results using real microbial networks

For both the mice gut microbiota and the *Ircinia oros* core microbiota presented in Fig. 6 of the main text, we selected a controlled GLV model to produce the simulation because it was used as the basis for the network inference algorithms (see the ecological networks in Fig. 3 of the main text). More precisely, with continuous control, the controlled GLV model is obtained from the general model by choosing

$$f(x) = \mathsf{diag}(x)[Ax + r], \quad g(x) = \mathsf{diag}(x)B,$$

while for impulsive control reads

$$f(x) = \mathsf{diag}(x)[Ax + r], \quad g(x) = B.$$

In both models, the zero/non-zero pattern of these two matrices A and B is determined by the controlled ecological network.

node	OTU number in *Ircinia oros* core microbiota	Species in mice gut microbiota
1	001801	*R. mirabilis*
2	001400	*B. ovalus*
3	001048	*B. vulgatus*
4	000669	*R. hominis*
5	000555	*P. dislasonis*
6	000271	*A. muciniphila*
7	290392	*C. difficile*
8	288080	*B. fragilis*
9	287579	*K. oxytoca*
10	238548	*C. ramnosum*
11	235338	*E. coli*
12	112902	*R. obeum*
13	107308	*C. scindens*
14	091937	*C. hiranonis*
15	042838	—
16	031796	—
17	009959	—
18	003837	—
19	002329	—
20	001995	—

Supplementary Table 1. **Correspondence between state nodes and species/OTUs of Fig. 3 in the main text.** OTUs as in [7] correspond to the state nodes in the *Ircinia oros* core microbiota network. Species as in [15] corresponding to nodes in the network of mice gut microbiota infected by *C. difficile*.

7.1. **Simulation results for the core microbiota of Ircinia oros.** The network inferred in [7], with 20 species (Table 1), was used to determine the zero-pattern of the A matrix. Its non-zero terms were

(approximately) chosen to fit the reported strength and sign of the interactions, leading to:

$$A = \begin{pmatrix}
0 & 0 & 0 & 0 & 0 & 0 & 0 & 0 & 0 & 0 & 0 & 0 & 0 & 0.12 & 0 & 0 & 0 & 0 & 0 & 0 \\
0 & -0.42 & 0 & 0 & 0 & 0 & 0 & 0 & 0 & 0 & 0 & 0 & 0 & 0 & 0 & 0 & 0 & 0 & 0 & 0 \\
0 & 0 & 0 & 0 & 0 & 0 & 0 & 0 & 0 & 0 & 1.5 & 0 & 0 & 0 & 0 & 0 & 0 & 0 & 0 & 0 \\
0 & 0 & 0 & 0 & 0 & 0 & 0 & 0 & 0 & 0 & 0 & 0 & 0 & 0 & 0 & -0.45 & 0 & 0 & 0 & 0 \\
0 & 0 & 0 & 0 & 0 & 0 & 0 & 0 & 0 & 0 & 0 & 0 & 0 & 0.11 & 0 & 0 & 0 & 0 & -0.15 & 0 \\
0 & 0 & 0 & 0 & 0 & 0 & 0 & 0 & 0 & 0 & 0 & 0 & 0 & 0 & 0 & 0 & 0 & 0 & 0 & 0 \\
0 & 0 & 0.11 & 0 & 0 & 0 & 0 & 0 & 0 & 0 & 0 & 0 & 0 & 0 & 0 & 0.35 & 0 & 0 & 0 & 0 \\
0 & 0 & 0 & 0 & 0 & 0 & 0 & 0 & 0 & 0 & 0 & 0 & 0 & 0 & 0 & 0 & -0.11 & 0 & 0 & 0 \\
0 & 0 & 3.69 & 0 & 0 & 0.12 & 0 & 0 & 0 & 0 & 0 & 0 & 0 & 0 & 0 & -0.21 & 0.11 & 0 & 0 & 0 \\
0 & 0 & 0 & 0 & 0 & 0 & 0 & 0 & 0 & 0 & 0 & 0 & 0 & 0 & -0.14 & 0 & 0 & 0 & 0 & 0 \\
0 & 0 & 0 & 0 & 0 & 0 & 0 & 0 & 0 & 0 & 0 & 0 & 0 & 0 & 0 & 0 & 0 & 0 & 0 & 1.31 \\
0 & 0 & 0 & 0 & 0 & 0 & 0 & 0 & 0 & 0 & 0 & 0 & 0 & 0 & 0 & 0 & 0 & 0 & 0 & 0 \\
0 & 0 & 0 & 0 & 0 & 0 & 0 & 0 & 0 & 0 & 0 & 0 & -5.98 & 0 & 0 & 0 & 0 & 0 & 0 & 0 \\
0 & 0 & -2.51 & 0 & 0 & 0 & 0 & 0 & 0 & 0 & 0 & 0 & 0 & 0 & 0 & 0 & 0 & 0 & 0 & 0 \\
0 & 0 & 0 & 0 & 0 & 0 & 0 & 0 & 0 & 0 & 0 & 0 & 0 & 0 & 0 & -6.14 & 0 & 0 & 0 & 0 \\
0 & 0 & 0 & 0 & 0 & 0 & 0 & 0 & 0 & 0 & 0 & 0 & 0 & 0 & 0 & 0 & 0 & 0 & 0 & 0 \\
0 & 0 & 0 & 0 & 0 & 0 & 0 & 0 & 0 & 0 & 0 & 0 & 0 & 0 & 0 & 0 & 0 & 0 & 0 & 0 \\
0 & 0 & 0 & 0 & 0 & 0 & 0 & 0 & 0 & 0 & 0 & 0.36 & 0 & 0 & 0 & 0 & 0 & 0 & 0 & 0 \\
0 & 0 & 0 & 0 & 0 & 0 & 0 & 0 & 0 & 0 & 0 & 0 & 0 & 0 & 0 & 0 & 0 & 0 & 0 & 0 \\
0 & 0 & 0.37 & 0 & 0 & 0 & 0 & 0 & 0 & 0 & 0 & 0 & 0 & 0 & 0 & 0 & 0 & 0 & 0 & 0
\end{pmatrix}.$$

Note that $A \in \mathbb{R}^{14\times 14}$ does not have full rank, implying that the corresponding GLV model admits true multi-stability. Indeed, if x^* is an equilibrium of the cGLV model without control, then $x^* + \ker A$ is also an equilibrium.

A minimal set of driver species was obtained as described in Section 4, defining the zero-pattern of the B matrix. Its non-zero entries were uniformly randomly chosen from the interval $[-1, 1]$ leading to

$$B = \begin{pmatrix}
0. & 0. & 0. & 0. & 0. & 0. & 0. & 0. & 0. & 0. \\
1.15 & 0. & 0. & 0. & 0. & 0. & 0. & 0. & 0. & 0. \\
0. & 0. & 0. & 0. & 0. & 0. & 0. & 0. & 0. & 0. \\
0. & 0. & 0. & 0. & 0. & 0. & 0. & 0. & -1.49 & 0. \\
0. & 0. & 0. & 0. & 0. & 0. & 0. & 0. & 0. & 0. \\
0. & 1.40 & 0. & 0. & 0. & 0. & 0. & 0. & 0. & 0. \\
0. & 0. & 0. & 0. & 0. & 0. & 0. & 0. & 0. & 1.27 \\
0. & 0. & 0. & 0. & 0. & 0. & 0. & 0. & 0. & 0. \\
0. & 0. & 0. & 0. & 0. & 0. & 0. & 0. & 0. & 0. \\
0. & 0. & 0. & 0. & 0. & 0. & 0. & 0. & 0. & 0. \\
0. & 0. & 0. & 0. & 0. & 0. & 0. & 0. & 0. & 0. \\
0. & 0. & 1.42 & 0. & 0. & 0. & 0. & 0. & 0. & 0. \\
0. & 0. & 0. & 1.18 & 0. & 0. & 0. & 0. & 0. & 0. \\
0. & 0. & 0. & 0. & 0. & 0. & 0. & 0. & 0. & 0. \\
0. & 0. & 0. & 0. & 0. & 0. & 0. & 0. & 0. & 0. \\
0. & 0. & 0. & 0. & -1.19 & 0. & 0. & 0. & 0. & 0. \\
0. & 0. & 0. & 0. & 0. & -1.19 & 0. & 0. & 0. & 0. \\
0. & 0. & 0. & 0. & 0. & 0. & 0. & 0. & 0. & 0. \\
0. & 0. & 0. & 0. & 0. & 0. & -1.198 & 0. & 0. & 0. \\
0. & 0. & 0. & 0. & 0. & 0. & 0. & -1.15 & 0. & 0.
\end{pmatrix}$$

We considered a "diseased" initial state

$$\begin{aligned} x_0 \;=\; & (0.4066, 0.5492, 0.4721, 0.5000, 0.5924, 0.4164, 1.4685, 0.5672, 0.5011, 0.5382, \\ & 0.5901, 0.4707, 0.5289, 0.5382, 0.4966, 0.5202, 0.4827, 0.4844, 0.5672, 0.5783)^{\intercal}. \end{aligned}$$

in which one species is overabundant (blue) compared to the rest. The growth rate vector r of the GLV model was chosen to ensure this initial state was an equilibrium, leading to

$$\begin{aligned} r \;=\; & (-0.0652252, 0.232317, -0.885135, 0.23632, 0.027358, 0., -0.237912, 0.0542032, -1.74108, \\ & 0.0710623, -0.758838, 0., 3.16484, 1.18613, 3.19597, 0., 0., -0.172444, 0., -0.176742)^{\intercal}. \end{aligned}$$

For the desired state, we considered another equilibrium with a better balance of species

$$\begin{aligned} x_{\mathrm{d}} \;=\; & (0.3658, 0.5492, 0.4721, 1.5400, 0.1776, 0.4164, 0.5835, 1.1573, 1.8976, 1.5352, 0.5901, \\ & 0.4707, 0.5289, 0.5382, 0.4966, 0.5202, 0.4827, 1.4844, 0.5672, 0.5783)^{\intercal}. \end{aligned}$$

A feedback controller $u(t) = K[x(t) - x_{\mathrm{d}}]$ with continuous control was designed using the methodology of Section 6 with $\hat{A} = A$, $\hat{B} = B$, $Q = I$ and $R = I$. Note this linear controller aims to control

the nonlinear GLV equations, despite the linearization of this nonlinear model at the desired state does not coincide with $\hat{A}$ and $\hat{B}$. This yields the gain matrix $K =$

$$\left(\begin{array}{cccccccccccccccccccc}
0 & 0.7 & 0 & 0 & 0 & 0 & 0 & 0 & 0 & 0 & 0 & 0 & 0 & 0 & 0 & 0 & 0 & 0 & 0 & 0 \\
0.41 & 0 & 0.2 & 0 & 0.05 & 1.07 & 0 & 0.42 & 0.81 & 0.01 & 0.14 & 0 & 0 & 1.12 & -0.03 & 0 & 0.03 & 0 & -0.01 & 0.04 \\
0 & 0 & 0 & 0 & 0 & 0 & 0 & 0 & 0 & 0 & 0 & 1.23 & 0 & 0 & 0 & 0 & 0 & 1 & 0 & 0 \\
0 & 0 & 0 & 0 & 0 & 0 & 0 & 0 & 0 & 0 & 0 & 0 & 0.1 & 0 & 0 & 0 & 0 & 0 & 0 & 0 \\
-0.01 & 0 & 0.05 & 0.03 & 0 & 0 & -0.03 & 0 & 0.02 & -1 & 0.02 & 0 & 0 & -0.02 & 1.08 & -3.48 & 0 & 0 & 0 & 0 \\
-0.24 & 0 & -0.2 & 0 & -0.04 & -0.03 & 0 & 0.91 & -0.34 & -0.01 & -0.14 & 0 & 0 & -0.43 & 0.01 & 0 & -1.11 & 0 & 0.01 & -0.04 \\
-0.25 & 0 & -0.21 & 0 & 0.97 & 0.01 & 0 & 0 & 0.07 & 0 & -0.13 & 0 & 0 & 0.21 & 0 & 0 & 0.01 & 0 & -1.12 & -0.03 \\
0.84 & 0 & -7.55 & 0 & 0.25 & -0.03 & 0 & 0.05 & -0.48 & -0.02 & -7.96 & 0 & 0 & 1.27 & 0.02 & 0 & -0.04 & 0 & -0.03 & -4.37 \\
0 & 0 & 0 & -1 & 0 & 0 & 0 & 0 & 0 & -0.03 & 0 & 0 & 0 & 0 & 0.04 & 0.04 & 0 & 0 & 0 & 0 \\
0 & 0 & 0.05 & 0 & 0 & 0 & 1 & 0 & 0 & -0.03 & 0.02 & 0 & 0 & 0.01 & 0.03 & 0.03 & 0 & 0 & 0 & 0
\end{array}\right)$$

In the case of impulsive control $u(t_k) = K[x(t_k) - x_{\mathrm{d}}]$, we set $\tau = 1$ and choose $\hat{A} = A$, $\hat{B} = B$, $\hat{B} = B$,$Q = 9I$ and $R = I$. This yields the gain matrix $K =$

$$\left(\begin{array}{cccccccccccccccccccc}
0 & 0.73 & 0 & 0 & 0 & 0 & 0 & 0 & 0 & 0 & 0 & 0 & 0 & 0 & 0 & 0 & 0 & 0 & 0 & 0 \\
0.27 & 0 & 0.12 & 0 & 0.03 & 0.68 & 0 & 0.28 & 0.51 & 0.01 & 0.06 & 0 & 0 & 0.71 & -0.02 & 0 & 0 & 0 & 0 & 0 \\
0 & 0 & 0 & 0 & 0 & 0 & 0 & 0 & 0 & 0 & 0 & 0.68 & 0 & 0 & 0 & 0 & 0 & 0.56 & 0 & 0 \\
0 & 0 & 0 & 0 & 0 & 0 & 0 & 0 & 0 & 0 & 0 & 0 & 0 & 0 & 0 & 0 & 0 & 0 & 0 & 0 \\
0 & 0 & 0.01 & 0 & 0 & 0 & 0 & 0 & 0 & -0.12 & 0 & 0 & 0 & 0 & 0.14 & -0.83 & 0 & 0 & 0 & 0 \\
-0.17 & 0 & -0.14 & 0 & -0.03 & 0 & 0 & 0.66 & -0.24 & -0.01 & -0.07 & 0 & 0 & -0.3 & 0.01 & 0 & -0.79 & 0 & 0 & 0 \\
-0.18 & 0 & -0.11 & 0 & 0.7 & 0 & 0 & 0 & 0.04 & 0 & -0.05 & 0 & 0 & 0.14 & 0 & 0 & 0 & 0 & -0.79 & 0 \\
0.09 & 0 & -0.98 & 0 & 0.02 & 0 & 0 & 0 & -0.04 & 0 & -1.21 & 0 & 0 & 0.14 & 0 & 0 & 0 & 0 & 0 & -0.87 \\
0 & 0 & 0 & -0.64 & 0 & 0 & 0 & 0 & 0 & -0.04 & 0 & 0 & 0 & 0 & 0.05 & 0 & 0 & 0 & 0 & 0 \\
0 & 0 & 0.05 & 0 & 0 & 0 & 0.74 & 0 & 0 & -0.04 & 0.02 & 0 & 0 & 0 & 0.04 & 0 & 0 & 0 & 0 & 0
\end{array}\right)$$

7.2. **Simulation results for the mice gut microbiota.** We considered the ecological network inferred in [15] with 14 species as in Table 1. We used this network to set the zero pattern of the A matrix. We next randomly choose the non-zero entries of A, keeping the relative strength and sign of the interactions as originally reported. This lead us to the matrix:

$$A = \left(\begin{array}{cccccccccccccc}
0. & 0. & 0. & 0. & 0. & 0. & 0. & 0. & 0. & 0. & 0. & 0. & 0. & 0. \\
0. & 0. & 1.1 & 0. & 0. & 0. & 0. & 0. & 0.95 & 0. & 0. & 0. & 0. & 0.98 \\
0. & 0. & 0. & 0. & 0. & 0. & 0. & 0. & 0. & 0. & 0. & 0. & 0. & -1.9 \\
0. & 0. & 0. & 0. & 0. & 0. & 1. & 0. & 0. & 0. & 0. & 0. & 0. & 0.97 \\
0. & 0. & 0. & 0. & 0. & 0. & 0. & 0. & 1.1 & 0. & 0. & 0. & 0. & 0. \\
0. & 0. & 0. & 0. & 0. & 0. & 0.94 & 0. & 1.2 & 0. & 0. & 0. & 0. & 0. \\
0. & 0. & 0. & -1. & 0. & 0. & 0. & 0. & 0. & 0. & 0. & 0. & -0.99 & 0. \\
0. & 0. & 0. & 0. & 0. & 0. & 0. & 0. & 0. & 0. & 0. & -1.2 & 0. & 0. \\
-1.2 & 0. & 0. & 0. & 0. & 0. & 0. & 0. & 0. & 0. & 2.2 & 0. & 0. & 0.95 \\
0. & 0. & 0. & 0. & 0. & 0. & 0. & 0. & 0. & 0. & 0. & 0. & 0. & 1. \\
0. & 0. & -1.1 & 0. & 0. & 0. & 0. & 0. & 0. & 0. & 0. & 0. & 0. & 0. \\
0. & 0. & 0. & 0. & 0. & 0. & 0. & 0. & 0. & 0. & 0. & 0. & 0. & 0. \\
2.1 & 0. & 0. & 0. & 0. & 0. & 0. & 0. & 0. & 0. & 0. & 0.99 & 0. & 0. \\
0. & 0. & 2.1 & 0. & 0. & -2.1 & 0. & 2.2 & 0. & 0. & -2.1 & 0. & 0. & 0.
\end{array}\right)$$

Next we identified a minimal set of driver species as described in Section 4. This yields a set of five species (see main text) which set the zero pattern of the B matrix. The non-zero entries of this matrix were randomly chosen from the interval $[-1, 1]$ to obtain

$$B = \left(\begin{array}{ccccc}
0.7 & 0. & 0. & 0. & 0. \\
0. & -0.34 & 0. & 0. & 0. \\
0. & 0. & 0. & 0. & 0. \\
0. & 0. & 0. & 0. & 0. \\
0. & 0. & 0. & 0. & 0. \\
0. & 0. & -0.54 & 0. & 0. \\
0. & 0. & 0. & 0. & 0. \\
0. & 0. & 0. & 0. & 0. \\
0. & 0. & 0. & 0. & 0. \\
0. & 0. & 0. & 0.38 & 0. \\
0. & 0. & 0. & 0. & 0. \\
0. & 0. & 0. & 0. & -0.047 \\
0. & 0. & 0. & 0. & 0. \\
0. & 0. & 0. & 0. & 0.
\end{array}\right).$$

The initial state was chosen as a "diseased" one in which *C. difficile* is overabundant compared to the rest of the species:

$$\begin{aligned} x_0 &= (0.5664, 0.5004, 0.5492, 0.5395, 0.5703, 0.4399, 1.5035, \\ &\quad 0.5853, 0.4707, 0.5515, 0.4102, 0.5406, 0.4803, 0.5375)^\intercal. \end{aligned}$$

The growth rate vector was then chosen to ensured that the above diseased state was an equilibrium, leading to

$$\begin{aligned} r &= -A^{-1}x_0 = (0, -1.5571, 0.9947, -2.0445, -0.5321, -1.9722, 1.0222, \\ &\quad 0.6225, -0.7628, -0.5556, 0.6245, 0, -1.7054, -0.6232)^\intercal. \end{aligned}$$

We found that in this dynamic model all equilibria have the same abundance of *C. difficile*. We then choose the desired state as another equilibrium with a better balance of species:

$$\begin{aligned} x_\mathrm{d} &= (0.5664, 1.2993, 0.5492, 0.7009, 1.6392, 1.0561, 1.5035, \\ &\quad 1.1888, 0.4707, 1.3033, 0.4102, 0.5406, 0.3161, 0.5375)^\intercal. \end{aligned}$$

A feedback controller $u(t) = K[x(t) - x_d]$ with continuous control was designed using the methodology of Section 6, using $\hat{A} = A$, $\hat{B} = B$ and

$$Q = 2I + \mathsf{diag}(-1.5, 10, 0, 0, -1.5, 4, 0, 0, 0, 0, 0, 5, 0, 0), \quad R = \mathsf{diag}(1.8, 1, 0.5, 0.5, 0.5).$$

This leads to the gain matrix

$$K = \begin{pmatrix} 4.4 & -0.4 & 0.39 & 1.06 & -0.29 & 0.13 & -1.28 & -0.13 & -1.77 & 0 & -2.86 & 0.82 & 2.25 & -0.78 \\ 0.35 & -3.41 & -2.12 & -0.23 & 0.11 & 0.12 & 0.08 & -0.14 & -2 & 0 & -2.01 & -0.24 & -0.39 & -0.41 \\ -0.36 & 0.4 & -2.32 & 0.98 & 0.81 & -10.6 & -1.56 & 11.22 & 3.26 & 0.03 & 7.71 & -0.49 & 2.54 & 12.73 \\ 0.01 & 0 & 0.97 & -0.01 & -0.01 & -0.02 & -0.01 & 0.01 & -0.09 & 2 & -0.51 & -0.03 & -0.04 & 0.06 \\ -0.2 & -0.07 & 0.21 & -0.17 & -0.08 & -0.04 & 0 & 4.09 & -0.43 & 0 & -0.56 & -15.02 & -0.32 & -0.28 \end{pmatrix}.$$

A feedback controller $u(t_k) = K[x(t_k) - x_d]$ was also constructed as in Section 6, using $\tau = 1$, $\hat{A} = A$, $\hat{B} = B$ and

$$Q = 9I + \mathsf{diag}(0, 0, 0, 0, -8, 0, 0, 0, 0, 0, 0, 0, 0, 0), \quad R = I.$$

This yields the gain matrix

$$K = \begin{pmatrix} 1.42 & -0.02 & 0.24 & 0.35 & -0.05 & 0 & -0.32 & 0.01 & -0.37 & 0 & -0.68 & 0.19 & 0.68 & -0.19 \\ 0.01 & -1.81 & -1.49 & -0.35 & 0.14 & 0.01 & 0.1 & -0.01 & -1.82 & 0 & -2.45 & -0.33 & -0.63 & -0.29 \\ 0 & 0.01 & 0.89 & 0.18 & 0.07 & -1.83 & -0.67 & 1.83 & -0.47 & 0 & -0.54 & -0.42 & 0.25 & 0.67 \\ 0 & 0 & 1.25 & -0.02 & -0.02 & 0 & 0.01 & -0.04 & -0.14 & 1.74 & -0.78 & -0.03 & -0.08 & -0.07 \\ -0.01 & -0.05 & 0.68 & -0.16 & -0.19 & -0.04 & -0.23 & 2.89 & -0.86 & 0 & -1.33 & -11.06 & -0.3 & -0.91 \end{pmatrix}.$$

Note, again, that both linear controllers aim to control a nonlinear cGLV model.

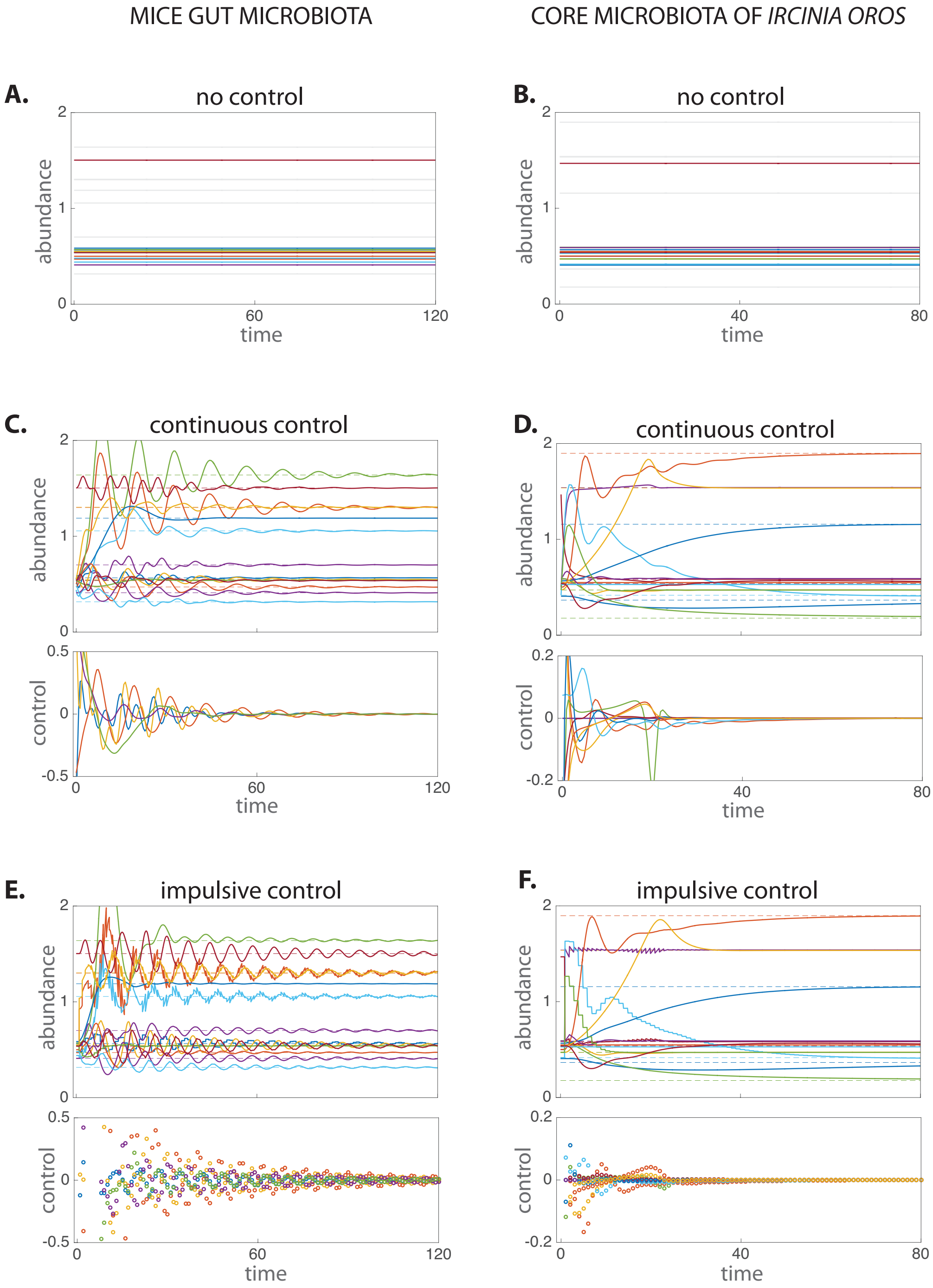

SUPPLEMENTARY FIGURE 1. **Controlling host-associated microbial communities. A, B.** Abundance of species in the microbial community without control (color). This corresponds to the initial state of the community x_0. One species (*C. difficile* in the case of the mice gut microbiota) is overabundant with respect to the rest. The desired state $x_{\rm d}$ is shown in grey. **C, D.** Control inputs obtained using the linear MPC for the continuous control scheme. These control inputs succeed in steering the state of the microbial community (solid) to the desired state (dashed). **E, F.** Control inputs obtained using the linear MPC for the impulsive control scheme. These control inputs succeed in steering the state of the microbial community (solid) to the desired state (dashed).

8. NUMERICAL VALIDATION IN LARGE MICROBIAL COMMUNITIES

To validate the proposed driver species and the linear MPC control method on large communities, we considered controlled population dynamics of the form

$$\dot{x} = \mathsf{diag}(x)[Af_\theta(x) + r], \quad \Delta x = Bu. \tag{S21}$$

Above, $A \in \mathbb{R}^{N\times N}$ and $B \in \mathbb{R}^{M\times N}$ are the interaction and susceptibility matrices of the community, $r \in \mathbb{R}^N$ is the intrinsic growth vector, and the function $f_\theta : \mathbb{R}^N \to \mathbb{R}^N$ is chosen as:

$$f_\theta(x_1, \cdots, x_N) = \left(\frac{x_1}{1+\theta_1 x_1}, \cdots, \frac{x_N}{1+\theta_N x_N}\right)^\intercal.$$

Note that f_0 correspond to Holling Type I functional responses, reducing Eq. (S21) to the GLV model. For $\theta \neq 0$, the function f_θ corresponds to Holling Type II functional responses.

As desired state $x_\mathrm{d} \in \mathbb{R}^N$, we chose an interior equilibrium of the community (i.e., a state where all species are present). This means that the control objective is to drive the community towards a state where all species coexist. We next generated random communities by choosing the parameters of Eq. (S21) as follows:

(1) First, an ecological network $\mathcal{G}(N, c)$ of a community was randomly generated using an Erdös-Rényi graph model for digraphs (without self-loops). In this model, N is the number of species and $c \in [0, 1]$ is the desired connectivity of the network. The edge-weights of this network were randomly chosen from a normal distribution $\mathcal{N}(0, \sigma)$, where $\sigma \geq 0$ captures the characteristic interspecies interaction strength. Second, a weighted adjacency matrix A_0 was calculated from $\mathcal{G}(N, c)$. Finally, we set $A = A_0 + \delta I_{N\times N}$, where $\delta = -1$ is the intraspecies interaction strength.

(2) The parameters $\theta = (\theta_1, \cdots, \theta_N)$ of the deformation were chosen uniformly at random from the interval $[0, \theta_{\max}]$, with $\theta_{\max} > 0$ a parameter to be selected.

(3) To ensure all randomly generated communities have x_d as interior equilibrium, we set $r = -Af_\theta(x_\mathrm{d})$.

(4) Based on generated ecological network $\mathcal{G}(N, c)$ a minimal set of driver species $\mathsf{X}_D \subseteq \{1, \cdots, N\}$ was identified according to Supplementary Note 4. We set $M = |\mathsf{X}_D|$ (i.e., the cardinality of X_D). For each driver species $j \in \mathsf{X}_D$, the corresponding column of the B matrix was chosen of the form $(0, \cdots, 0, b_j, 0, \cdots, 0)^\intercal$, where b_j (located in j-th row) was chosen uniformly at random from the interval $[0, 1]$.

(5) We choose equally spaced intervention instants $t_k \in \mathbb{T}$, where $t_{k+1} - t_k = \tau$ for $\tau = 0.1$. The linear MPC controller $u(t_k) = K[x(t_k) - x_\mathrm{d}]$ is calculated as in Supplementary Note 6. The matrices $Q = 2 \times 10^4 I$ and $R = 1.5 \times 10^{-1} I$ were used for all ecosystems.

The result of the above procedure are illustrated in the Supplementary Figure 2 for $N = 100$ species, $c = 0.04$, $\sigma = 0.7$, and $\theta_{\max} = 0.01$. For this community, we identified a minimal set of just three driver species $\mathsf{X}_D = \{36, 52, 60\}$. The linear impulsive MPC applied to these three driver species succeeds in driving the whole community to the desired state x_d (Supplementary Figure 2b,c). By contrast, the uncontrolled community does not reach the desired state and enters into a very oscillatory response where some species become extinct and others are overabundant (Supplementary Figure 2d). In other words, the community will not naturally evolve towards the desired state.

We used the above procedure to calculate the *success rate* $s_\varepsilon(d, N, c, \sigma, \theta_{\max})$ of our control framework, defined as the probability that the state $x(t)$ of a controlled random community with parameters $(N, c, \sigma, \theta_{\max})$, and that starts at distance d from x_d, is driven to an ε-neighborhood of x_d. Specifically, for each realization of a community with parameters $(N, c, \sigma, \theta_{\max})$, this probability was estimated as follows:

(6) Given the distance $d \geq 0$, we generate a set $X_0 \subseteq \mathbb{R}^N$ of k initial conditions sampling from $x_\mathrm{d} + d\,\eta$, where $\eta \in \mathbb{R}^N$ is a random vector with unit norm. If for some initial condition

$x_0 \in X_0$ the initial abundance of species i is negative $x_{0,i} < 0$, we reset its initial abundance as $x_{0,i} = 0.1$.

(7) Discard from X_0 all those initial conditions that naturally evolve to the desired state x_d. Specifically, we denote by $x_{NC}(x_0; T)$ the state of the uncontrolled community starting at x_0 at time T. Then, we only keep those $x_0 \in X_0$ such that $\|x_{NC}(x_0; T) - x_\mathrm{d}\|_\infty > \|x_0 - x_\mathrm{d}\|_\infty$ for $T = 10\tau$.

(8) For each remaining initial condition $x_0 \in X_0$, simulate the controlled population dynamics for a sufficiently large time $T > 0$. Let $x(x_0; T)$ denote the state of the controlled community at time T given it had x_0 as initial condition. Then, the success rate s_ε is calculated as

$$s_\varepsilon(d, N, C, \sigma, \theta_\mathrm{max}) = \frac{|\{x_0 \in X_0 : \|x(x_0; T) - x_\mathrm{d}\|_\infty \leq \varepsilon\}|}{|X_0|}.$$

Above, the symbol $|A|$ denotes the cardinality of the set A.

Finally, we calculate the *mean success rate* $\langle s_\varepsilon(d, N)\rangle$ as the average of $s_\varepsilon(d, N, c, \sigma, \theta_\mathrm{max})$ over 100 random communities with parameters $(c, \sigma, \theta_\mathrm{max})$. For the results in Fig. 5 of the main text, we used $N = 100$, $T = 700$, $k = 300$ and $\varepsilon = 10^{-2}$. In the `DriverSpeciesModule` Julia package, the function `GetControllerSucess_Rate_OverNetworkRealizations` implements the above procedure.

Next, to introduce errors into the ecological network used to identify the sensor species and to build the linear MPC, we define a rewiring probability $p \in [0, 1]$. The end-node of each edge in the network $\mathcal{G}(N, c)$ is rewired with probability p, choosing the new end-node uniformly among all nodes in the network. This is implemented as the `RewireNetwork` function in the `DriverSpeciesModule` Julia package.

The `DriverSpeciesModule` package is available at GitHub address:

`https://github.com/mtangulo/DriverSpecies`

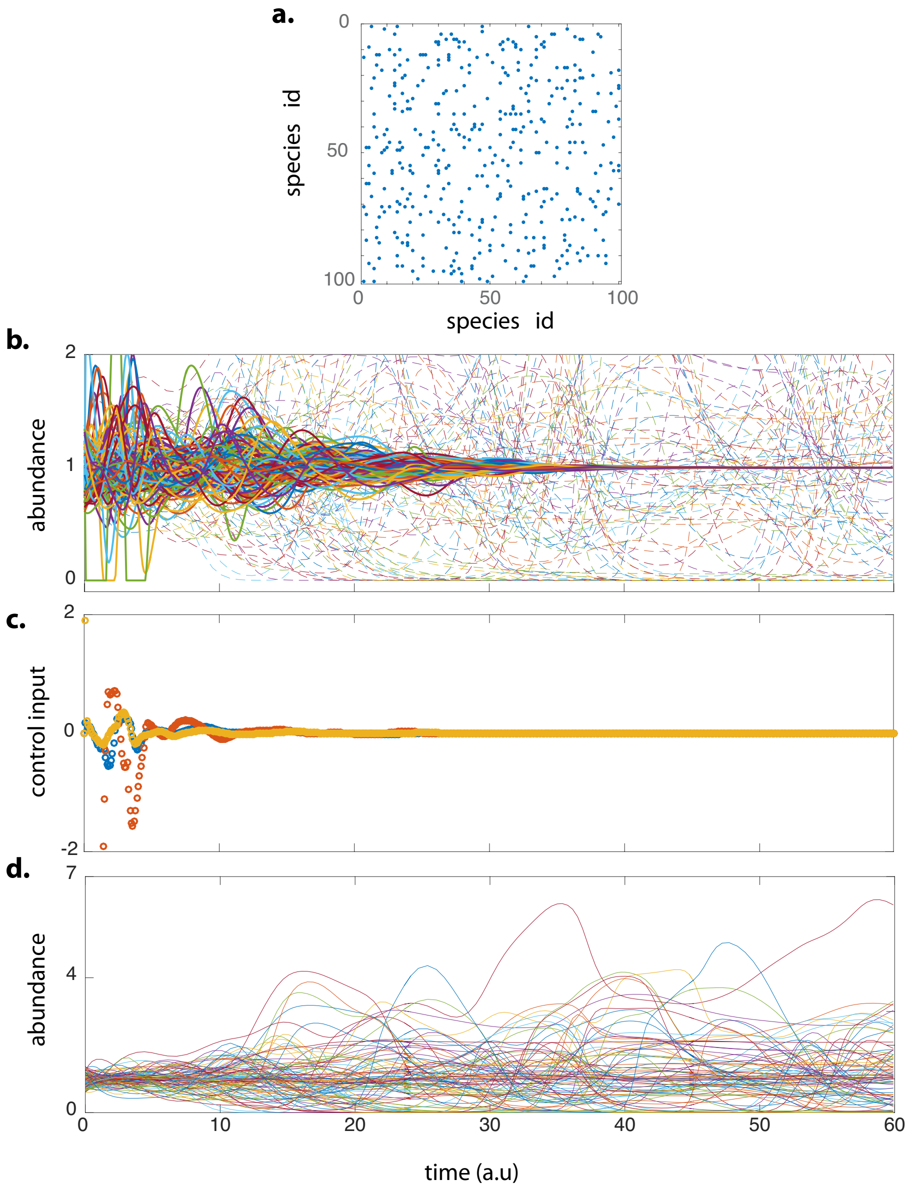

SUPPLEMENTARY FIGURE 2. **Controlling a large community.** Simulation results on a random microbial community of $N = 100$ species with network parameters $c = 0.04$ and $\sigma = 0.7$. The desired state is chosen as $x_d = (1, \cdots, 1)^\mathsf{T}$. The rest of the parameters are $\tau = 0.1$ and $\theta_{\max} = 0.01$. Based on the ecological network of this community, we identified a minimal set of driver species with three elements $\mathsf{X}_D = \{36, 52, 60\}$. **a.** Matrix plot of the adjacency matrix A_0 of the ecological network of the community. The network contains 405 interactions. **b.** The initial condition of the community x_0 is chosen randomly as $x_0 = x_\mathrm{d} + d\eta$, where the distance is $d = 0.4$. The controlled community is driven to the desired state (solid lines) using the linear MPC applied to the three driver species. Notice that control action remove and the reintroduce some driver species. By contrast, the uncontrolled community does not reach the desired state, and some of its species become extinct (dashed lines). **c.** Impulsive control strategy applied to the three driver species generated by the linear MPC method. **d.** Trajectories of the uncontrolled community. The community does not reach the desired state, but enters into a rather complex temporal response.

9. Controlling the repressilator

We consider the repressilator with the parametrization and assumptions used in [36]. In such case, its biochemical dynamics take the form

$$\dot{x}_1 = \frac{\alpha}{1+x_2^p} - x_1, \quad \dot{x}_2 = \frac{\alpha}{1+x_3^p} - x_2, \quad \dot{x}_3 = \frac{\alpha}{1+x_1^p} - x_3,$$

where x_i is proportional to the protein concentration, $\alpha > 0$ represents the gene expression from the promoters, and $p > 0$ characterizes the form of the saturation. The repressilator has been shown to produce sustained oscillations for $p > 2$ and large enough α. For the results we present, we consider the parameters $\alpha = 4$ and $p = 3$ that produce sustained oscillations. Also, these parameters yield the following equilibrium:

$$x_{\mathrm{d}} = (1.2837, 1.2837, 1.2837)^{\intercal}.$$

Next we aim to "kill" the oscillations in the repressilator by driving its state to x_d. The underlying network of the repressilator suggest that it is sufficient to actuate one of its three proteins, because that will provide a path that covers all nodes (Supplementary Figure 2A). We thus consider one control input u_1 impulsively actuating one protein (without loss of generality we choose x_3), leading to the equation:

$$x_3(t^+) = x_3(t) + u_1(t), \quad t \in \mathbb{T} = \{0, 1, 2, \cdots\}.$$

To test for the absence of autonomous elements, we compute the corresponding sequence of subspaces $\mathcal{H}_k$'s to obtain

$$\mathcal{H}_1 = \operatorname*{span}_{\mathcal{K}}\{\mathrm{d}x_1, \mathrm{d}x_2, \mathrm{d}x_3\}, \quad \mathcal{H}_2 = \operatorname*{span}_{\mathcal{K}}\{\mathrm{d}x_1, \mathrm{d}x_2\}, \quad \mathcal{H}_3 = \operatorname*{span}_{\mathcal{K}}\{\mathrm{d}x_1\}, \quad \mathcal{H}_4 = \{0\}.$$

According to Theorem 2, actuating x_3 is enough to eliminate all autonomous elements. Therefore, $\{\mathsf{x}_3\}$ alone is a "driver protein".

To design the control actions to be applied to the above driver protein, notice that $\mathcal{H}_3$ is integrable. Therefore, according to Theorem 5, this implies that an impulsive (nonlinear) MPC with a prediction horizon of $L = 3$ is sufficient to steer the system to x_{d}. We calculated this MPC strategy from the initial state $x(0) = (1.2742, 1.0297, 1.6544)^{\intercal}$ obtaining

$$u(t_1) = -0.4536, \quad u(t_2) = 0.2283, \quad u(t_3) = 5.3518 \times 10^{-9},$$

as shown in Supplementary Figure 3A. The calculated MPC control strategy can drive the represilator to x_{d} with a precision of 1.0184×10^{-13}, as shown in Supplementary Figure 3B. This figure also compares the effectivity of the MPC control strategy with respect to the behavior of the repressilator without control.

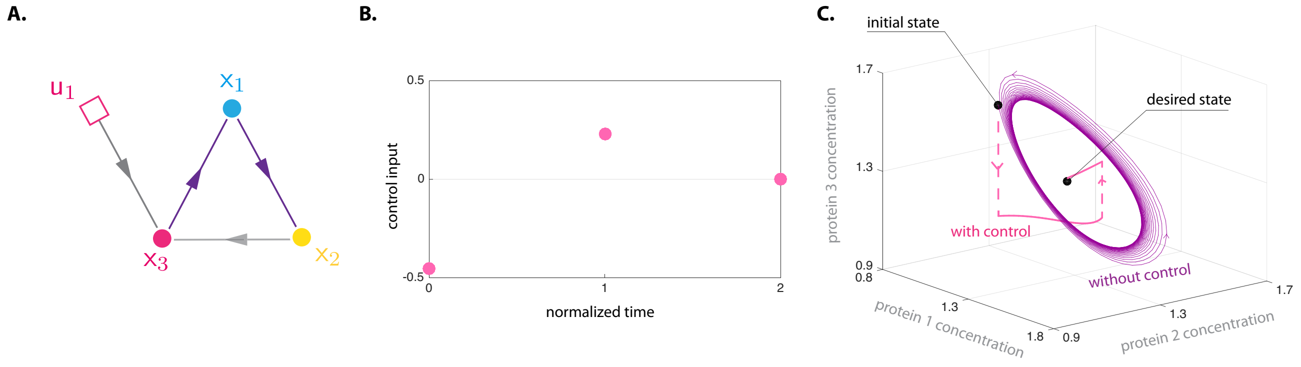

SUPPLEMENTARY FIGURE 3. **Controlling the repressilator.** **A.** Network underlying the repressilator with each node representing a protein. **B.** Control actions obtained by using MPC on the driver protein. **C.** Trajectories of the repressilator with and without control. The initial state is $x(0) = (1.2742, 1.0297, 1.6544)^{\intercal}$.

SUPPLEMENTARY REFERENCES

[1] Jack A Gilbert, Joshua A Steele, J Gregory Caporaso, Lars Steinbrück, Jens Reeder, Ben Temperton, Susan Huse, Alice C McHardy, Rob Knight, Ian Joint, et al. Defining seasonal marine microbial community dynamics. *The ISME journal*, 6(2):298–308, 2012.

[2] Lutz Becks, Frank M Hilker, Horst Malchow, Klaus Jürgens, and Hartmut Arndt. Experimental demonstration of chaos in a microbial food web. *Nature*, 435(7046):1226–1229, 2005.

[3] Stefanie Widder, Rosalind J Allen, Thomas Pfeiffer, Thomas P Curtis, Carsten Wiuf, William T Sloan, Otto X Cordero, Sam P Brown, Babak Momeni, Wenying Shou, et al. Challenges in microbial ecology: building predictive understanding of community function and dynamics. *The ISME journal*, 2016.

[4] Sonia R Vartoukian, Richard M Palmer, and William G Wade. Strategies for culture of 'unculturable' bacteria. *FEMS microbiology letters*, 309(1):1–7, 2010.

[5] Jonathan Friedman, Logan M Higgins, and Jeff Gore. Community structure follows simple assembly rules in microbial microcosms. *Nature Ecology & Evolution*, (1):0109, 2017.

[6] Vanni Bucci, Belinda Tzen, Ning Li, Matt Simmons, Takeshi Tanoue, Elijah Bogart, Luxue Deng, Vladimir Yeliseyev, Mary L Delaney, Qing Liu, et al. Mdsine: Microbial dynamical systems inference engine for microbiome time-series analyses. *Genome biology*, 17(1):121, 2016.

[7] Torsten Thomas, Lucas Moitinho-Silva, Miguel Lurgi, Johannes R Björk, Cole Easson, Carmen Astudillo-García, Julie B Olson, Patrick M Erwin, Susanna López-Legentil, Heidi Luter, et al. Diversity, structure and convergent evolution of the global sponge microbiome. *Nature Communications*, 7, 2016.

[8] Yandong Xiao, Marco Tulio Angulo, Jonathan Friedman, Matthew K Waldor, Scott T Weiss, and Yang-Yu Liu. Mapping the ecological networks of microbial communities. *Nature communications*, 8(1):2042, 2017.

[9] Hyun-Seob Song, William R Cannon, Alexander S Beliaev, and Allan Konopka. Mathematical modeling of microbial community dynamics: a methodological review. *Processes*, 2(4):711–752, 2014.

[10] Mark Vellend and Anurag Agrawal. Conceptual synthesis in community ecology. *The Quarterly review of biology*, 85(2):183–206, 2010.

[11] Weigang Hu, Qi Zhang, Tian Tian, Dingyao Li, Gang Cheng, Jing Mu, Qingbai Wu, Fujun Niu, James C Stegen, Lizhe An, et al. Relative roles of deterministic and stochastic processes in driving the vertical distribution of bacterial communities in a permafrost core from the qinghai-tibet plateau, china. *PloS one*, 10(12):e0145747, 2015.

[12] John C Moore, Peter C de Ruiter, H William Hunt, David C Coleman, and Diana W Freckman. Microcosms and soil ecology: critical linkages between fields studies and modelling food webs. *Ecology*, 77(3):694–705, 1996.

[13] Jérôme Mounier, Christophe Monnet, Tatiana Vallaeys, Roger Arditi, Anne-Sophie Sarthou, Arnaud Hélias, and Françoise Irlinger. Microbial interactions within a cheese microbial community. *Applied and environmental microbiology*, 74(1):172–181, 2008.

[14] Karoline Faust and Jeroen Raes. Microbial interactions: from networks to models. *Nature Reviews Microbiology*, 10(8):538–550, 2012.

[15] Richard R Stein, Vanni Bucci, Nora C Toussaint, Charlie G Buffie, Gunnar Rätsch, Eric G Pamer, Chris Sander, and João B Xavier. Ecological modeling from time-series inference: insight into dynamics and stability of intestinal microbiota. *PLoS Comput Biol*, 9(12):e1003388, 2013.

[16] Georg K Gerber. The dynamic microbiome. *FEBS letters*, 588(22):4131–4139, 2014.

[17] Charlie G Buffie, Vanni Bucci, Richard R Stein, Peter T McKenney, Lilan Ling, Asia Gobourne, Daniel No, Hui Liu, Melissa Kinnebrew, Agnes Viale, et al. Precision microbiome reconstitution restores bile acid mediated resistance to clostridium difficile. *Nature*, 517(7533):205–208, 2015.

[18] Katharine Z Coyte, Jonas Schluter, and Kevin R Foster. The ecology of the microbiome: Networks, competition, and stability. *Science*, 350(6261):663–666, 2015.

[19] Travis E Gibson, Amir Bashan, Hong-Tai Cao, Scott T Weiss, and Yang-Yu Liu. On the origins and control of community types in the human microbiome. *PLoS Comput Biol*, 12(2):e1004688, 2016.

[20] Amir Bashan, Travis E Gibson, Jonathan Friedman, Vincent J Carey, Scott T Weiss, Elizabeth L Hohmann, and Yang-Yu Liu. Universality of human microbial dynamics. *Nature*, 534(7606):259–262, 2016.

[21] Phuongan Dam, Luis L Fonseca, Konstantinos T Konstantinidis, and Eberhard O Voit. Dynamic models of the complex microbial metapopulation of lake mendota. *NPJ Systems Biology and Applications*, 2:16007, 2016.

[22] Christian Jost and Stephen P Ellner. Testing for predator dependence in predator-prey dynamics: a non-parametric approach. *Proceedings of the Royal Society of London B: Biological Sciences*, 267(1453):1611–1620, 2000.

[23] Vanni Bucci and Joao B Xavier. Towards predictive models of the human gut microbiome. *Journal of molecular biology*, 426(23):3907–3916, 2014.

[24] Giuseppe Conte, Claude H Moog, and Anna Maria Perdon. *Algebraic methods for nonlinear control systems*. Springer Science & Business Media, 2007.

[25] E Aranda-Bricaire, CH Moog, and J-B Pomet. A linear algebraic framework for dynamic feedback linearization. *IEEE Transactions on Automatic Control*, 40(1):127–132, 1995.

[26] Xinzhi Liu. Impulsive control and optimization. *Applied mathematics and computation*, 73(1):77–98, 1995.

[27] Tao Yang. *Impulsive control theory*, volume 272. Springer Science & Business Media, 2001.

[28] Hirokazu Mayeda. On structural controllability theorem. *IEEE Transactions on Automatic Control*, 26(3):795–798, 1981.

[29] Sergio Daniel Pequito, Soummya Kar, A Pedro Aguiar, et al. A framework for structural input/output and control configuration selection in large-scale systems. *IEEE Trans. Automat. Contr.*, 61(2):303–318, 2016.

[30] Jean-Michel Dion, Christian Commault, and Jacob Van Der Woude. Generic properties and control of linear structured systems: a survey. *Automatica*, 39(7):1125–1144, 2003.

[31] Peter Dorato, Vito Cerone, and Chaouki Abdallah. *Linear quadratic control: an introduction*. Krieger Publishing Co., Inc., 2000.

[32] Karl Johan Aström and Richard M Murray. *Feedback systems: an introduction for scientists and engineers*. Princeton university press, 2010.

[33] Brian DO Anderson and John B. Moore. *Optimal control: linear quadratic methods*. Courier Corporation, 2007.

[34] U Shaked. Guaranteed stability margins for the discrete-time linear quadratic optimal regulator. *IEEE Transactions on Automatic Control*, 31(2):162–165, 1986.

[35] Isaac Klickstein, Afroza Shirin, and Francesco Sorrentino. Locally optimal control of complex networks. *Physical review letters*, 119(26):268301, 2017.

[36] Olguta Buse, Rodrigo Pérez, and Alexey Kuznetsov. Dynamical properties of the repressilator model. *Physical Review E*, 81(6):066206, 2010.
